# Human mesenchymal stroma/stem-like cell-derived taxol-loaded EVs/exosomes transfer anti-tumor microRNA signatures and express enhanced SDF-1-mediated tumor tropism

**DOI:** 10.1186/s12964-024-01886-2

**Published:** 2024-10-17

**Authors:** Ralf Hass, Juliane von der Ohe, Tianjiao Luo

**Affiliations:** Department of Obstetrics and Gynecology, Biochemistry and Tumor Biology Laboratory, Hannover Medical School, 30625 Hannover, Germany

**Keywords:** Mesenchymal stroma cells, Chemotherapeutics, Extracellular vesicles, Exosomes, Tumor plasticity, Tumor tropism, Stroma cell-derived factor, microRNAs

## Abstract

**Background:**

The release of extracellular vesicles (EVs) including exosomes from human mesenchymal stroma/stem-like cells (MSC) represents valuable cell-free carriers for the delivery of regenerative and medicinal compounds.

**Methods:**

EVs/exosomes were isolated by differential centrifugation from four individual MSC as controls and after treatment with a sub-lethal concentration of 10 mM taxol for 24 h, respectively. The isolated EVs/exosomes were characterized and quantified by nano-tracking-analysis and by Western blots. MicroRNAs (miRs) were isolated from the different EVs/exosome populations and expression levels were quantified by qPCR using 1246 miR templates. Cytotoxic effects of the different MSC-derived taxol-loaded EVs/exosomes were determined in five different GFP-transduced cancer cell lines and quantified by a fluoroscan assay with a GFP-detecting fluorimeter. The presence of stroma cell-derived factor 1 (SDF-1) in MSC-derived EVs/exosomes and its enhanced expression in the vesicles after taxol treatment of MSC was quantified by a specific ELISA.

**Results:**

EVs/exosomes isolated from four individual taxol-treated MSC displayed a larger size and higher yields as the control EVs/exosomes and were used as anti-tumor therapeutic vehicles. Application of each of the four MSC-derived taxol-loaded EVs/exosome populations revealed significant cytotoxic effects in cell lines of five different tumor entities (carcinomas of lung, breast, ovar, colon, astrocytoma) in a concentration-dependent manner. Expression analysis of 1246 miRs in these taxol-loaded EVs/exosomes as compared to the corresponding MSC-derived control EVs/exosomes unraveled a taxol-mediated up-regulation of 11 miRs with predominantly anti-tumorigenic properties. Moreover, various constitutively expressed protein levels were unanimously altered in the MSC cultures. Taxol treatment of the different MSC revealed an up-regulation of tetraspanins and a 2.2-fold to 5.4-fold increased expression of SDF-1 among others. Treatment of cancer cells with MSC-derived taxol-loaded EVs/exosomes in the presence of a neutralizing SDF-1 antibody significantly abolished the cytotoxic effects between 20.3% and 27%.

**Conclusions:**

These findings suggested a taxol-mediated increase of anti-cancer properties in MSC that enhance the tropism of derived EVs/exosomes to tumors, thereby specifically focusing the therapeutic effects of the delivered products.

**Supplementary Information:**

The online version contains supplementary material available at 10.1186/s12964-024-01886-2.

## Background

Tissue-derived primary human mesenchymal stroma/stem-like cells (MSC) represent a multipotent but heterogeneous compilation of interdependent types of distinct stroma and stem-like cells rather than a uniform population. These cells predominantly reside in perivascular regions of various organs and tissues [1, 2] and should be discriminated from fibroblasts or pericytes [3]. MSC can also be retrieved from neonatal tissues. In particular, MSC from umbilical cord tissue provide a rich source of naïve stem cells with superior growth and expansion capacity as compared to MSC from adult sources like bone marrow or adipose tissues [4].

Physiological functions of MSC include repair and regenerative activities and various clinical studies are using MSC to treat hematological diseases, graft-versus-host disease, organ transplantation, diabetes, inflammatory diseases, bone and cartilage, neurological, and skin diseases, among others [5–8]. MSC can also play a significant role during tumor development by various types of cellular communication processes with cancer cells [9]. Eventually, MSC can also fuse with cancer cells in vitro [10] and in vivo [11] by the formation of new cancer hybrid cells [12, 13] displaying increased tumor plasticity associated with completely new tumorigenic and metastatic properties [14, 15].

These MSC-mediated interactions are accompanied by the release of trophic factors and extracellular vesicles (EVs). MSC-mediated secretion of trophic compounds include among others various immune-modulatory substances such as prostaglandin E2, growth factors, and multiple chemokines such as C-X-C motif ligand 12 (CXCL12) also known as stroma cell-derived factor-1 (SDF-1). Previous studies have suggested that SDF-1 can perform receptor-mediated interactions e.g. by binding to its specific receptors CXCR4 and CXCR7 on cancer cells, thereby contributing to cancer cell proliferation, angiogenesis, invasion, and tumor metastasis [16].

MSC-mediated release of EVs such as microvesicles and exosomes can transport cargo to interacting recipient cells. Previous work in a human MSC-like cell line [17] has demonstrated that isolated fractions of microvesicles display different markers and biological effects as compared to the much smaller exosomes [18]. Exosomes represent small particles of approximately 20 to 200 nm in diameter, which arise as multivesicular bodies of endocytic origin released into the extracellular compartment [19]. These small particles can be identified by typical marker proteins including surface glycoproteins of the tetraspanin transmembrane-4 family such as CD9, CD63, and CD81 (= TAPA-1 (target of the antiproliferative antibody 1) = tetraspanin-28) [20, 21]. Nearly all cell types can produce exosomes including cancer cells. Therefore, exosomes can also change significantly during disease, inflammation, and cancer [22]. Accordingly, questions remained as to whether MSC-mediated addressing and transport of trophic factors such as SDF-1 and further compounds via exosomes may contribute to enhanced interactions with cancer cells and increased tropism versus tumors.

According to the large amount of released trophic compounds MSC-derived exosomes are gaining increased attention for potential clinical applications as a cell-free system of vesicular products [23]. Among further signaling molecules exosomes also contain a variety of different RNAs such as mRNAs, long non-coding RNAs, and regulatory microRNAs (miRs), which can alter the functionality of recipient cells [21, 24]. In particular, exosome-transported miRs are epigenetically involved in gene expressions and associated with the regulation of multiple cellular pathways in target cells. Each miR is predicted to regulate hundreds of biological targets whereby the majority of protein-coding genes is thought to be under their control [25]. In practice, this means that miR-dependent regulation is involved in various metabolic pathways.

Structurally, miRs represent small non-coding single-stranded RNAs of about 22 nucleotides, which regulate gene expression by predominantly binding to the 3'-untranslated region (3'-UTR) and, to a lesser extend, to the 5'-untranslated region (5'-UTR) of mRNA [26–28]. The biogenesis of miRs includes a predominant canonical and further alternative pathways. Basically, primary miRs (pri-miRs) are formed after transcription from the DNA. These precursor miRs are processed into mature miRs by different steps of cleavage and conformational changes with enzyme complexes, including RNA binding protein DGCR8, the ribonuclease Drosha and the endonuclease Dicer [29]. In certain neoplastic developments miRs can be dysregulated. Their dysregulation may be involved in tumor initiation and progression [30]. Dysregulated miRs have been shown to affect the hallmarks of cancer, including sustaining proliferative signaling, evading growth suppressors, resisting cell death, and activating invasion and metastasis [31]. Mature miRs can be secreted into the circulation or shuttled via exosomes among different cells and may therefore also serve as diagnostic tools for potential biomarkers of various (cancerous) diseases. However, it remains unclear, how changes to the cellular microenvironment such as exposure of MSC to taxol may affect alterations in the miR pattern. At least, the profound influences and critical regulations of many biological processes by miRs include growth, differentiation, apoptosis, motility and malignant transformation. Moreover, depending on exogenous conditions miRs can function as either oncogenes or tumor suppressors.

The present study unravels distinct therapeutic properties of taxol-treated MSC-derived exosomes with the expression of predominant anti-tumor miR patterns and increased tumor tropism via an elevated SDF-1/CXCR4/CXCR7 axis.

## Methods

### Cell culture

The isolation of primary human MSC from umbilical cord tissue has been approved by the Ethics Committee of Hannover Medical School, Project #443 on February 26th, 2009. Informed written consent was obtained from all donors.

Briefly, primary human MSC were isolated from umbilical cord explant cultures as reported previously [32]. The cells were cultured in αMEM (Sigma Chemie GmbH, Taufkirchen, Germany) supplemented with 10% of allogeneic human AB-serum (Pan Biotech GmbH, Aidenbach, Germany), 100U/ml penicillin, 100 µg/ml streptomycin and 2 mM L-glutamine (Sigma). Subculture of MSC was performed following accutase (Sigma) treatment for 3 min at 37 °C. The characteristics for MSC were tested as extensively outlined previously [33, 34]. Primary MSC from five different donors and passages (MSC241111 P4; MSC280416 P6; MSC180314 P2; MSC270815 P4 were used in the experiments and the human cell line MSC544 as described elsewhere [17].

Primary cultures of normal human mammary epithelial cells (HMEC) were commercially obtained from BioWhittaker Inc. (Walkersviell, MD, USA) (Lot #1F1012). The HMEC were cultured as described previously [35] in serum-free mammary epithelial cell growth medium (MEBM) (PromoCell GmbH, Heidelberg, Germany) supplemented with 52 µg/ml of bovine pituary extract, 0.5 µg/ml of hydrocortisone, 10 ng/ml of human recombinant epidermal growth factor and 5 µg/ml of human recombinant insulin (all from PromoCell GmbH) in a humidified atmosphere at 37 °C. The HMEC were transduced with a lentiviral GFP-vector according to a previous method for labeling primary cells [36] and used as HMEC^GFP^ in passage 13.

Human SK-OV-3 ovarian cancer cells were commercially obtained in P25 from American Type Culture Collection (ATCC), Manassas, VA, USA (ATCC® #HTB-77TM) originally established from the malignant ascites of a patient with progressive adenocarcinoma of the ovary. The ovarian cancer cells were cultivated in RPMI 1640 medium supplemented with 10% (v/v) fetal calf serum, 2 mM L-glutamine, 100U/ml penicillin and 100 µg/ml streptomycin (all from Sigma).

Human A549 lung carcinoma and human triple negative MDA-MB-231 breast carcinoma cells were used as reported previously [37, 38]. The lung carcinoma populations were cultivated at 1,750 cells/cm^2^ in RPMI 1640 medium supplemented with 10% (v/v) fetal calf serum, 2 mM L-glutamine, 100U/ml penicillin and 100 µg/ml streptomycin (all from Sigma). Culture of the breast carcinoma cells was performed using Leibowitz L-15 medium supplemented with 10% (v/v) fetal calf serum, 2 mM L-glutamine, 100U/ml penicillin and 100 µg/ml streptomycin (all from Sigma). Human HT-29 colon adenocarcinoma cells were cultured in McCoy's 5A medium (Sigma) supplemented with 100U/ml penicillin, 100 µg/ml streptomycin, 2 mM L-glutamine (Sigma), and 10% (v/v) FCS (Pan Biotech GmbH).

Human CCF-STTG1 astrocytoma cells were commercially obtained from ATCC (ATCC® #CRL-1718™). This cell line was originally established from a grade IV astrocytoma of a 68-year-old female. The cells were cultured in DMEM (Sigma) supplemented with 100U/ml penicillin, 100 µg/ml streptomycin and 2 mM L-glutamine (Sigma) and 10% (v/v) FCS (Pan Biotech GmbH).

Subculture of all cancer cell lines was performed by trypsin/EDTA (Biochrom GmbH, Berlin, Germany) treatment for 5 min at 37 °C.

The cell lines were tested for mycoplasma by the luminometric MycoAlert Plus mycoplasma detection kit (Lonza Inc., Rockland, ME, USA) according to the manufacturer’s recommendations. Cell line authentication was performed by short tandem repeat (STR) fragment analysis using the GenomeLab human STR primer set (Beckman Coulter Inc., Fullerton, CA, USA) and was confirmed in previous work [39] according to the STR database provided by the ATCC, Manassas, VA, USA.

In order to demonstrate cellular interactions between MDA-MB-231^cherry^ and MSC060616^GFP^ P4 and to quantify cytotoxic effects of the exosomes in the fluoroscan assay, the corresponding cell populations were transduced with a 3rd generation lentiviral SIN vector containing either the mcherry or the eGFP gene as described in previous work [40].

### Preparation of control exosomes and taxol-loaded exosomes

Subconfluent MSC cultures (about 10^6^ cells) at a density of 7 × 10^3^ cells/cm^2^ were incubated either in appropriate dilution (1:700) of solvent (49.7% ethanol = final concentration of 0.07% ethanol) for control exosomes or in the presence of 10 µM taxol (paclitaxel in solvent, diluted 1:700 from a 7 mM stock solution) (Fresenius Kabi Deutschland GmbH, Bad Homburg, Germany) for 24 h, washed three times with serumfree culture medium, and incubated with serumfree culture medium for further 24 h. Thereafter, the conditioned medium was removed and used for EV/exosome preparations by three different methods.

The differential ultracentrifugation method was performed in four subsequent centrifugation steps (1.step: 360 g for 10 min to remove cells; 2.step: 2,000 g for 10 min to remove dead cells; 3.step: 10,000 g for 30 min to remove debris and large vesicles; 4.step: 100,000 g for 70 min to precipitate exosome-like particles) according to the protocol by Thery et al. [41].

Alternatively, a size exclusion chromatography (SEC) method was applied. The conditioned medium from taxol-loaded MSC was concentrated about 100-fold in an Amicon® Ultra-15 centrifugal filter device (Millipore/Merck/Sigma-Aldrich Chemie GmbH, Taufkirchen, Germany), (molecular weight cutoff 100 kDA) equilibrated with αMEM medium at 3,200 g for 25 min. The concentrated supernatant of about 250 µl was then applied to a PURE-EVs column (HansaBioMed Life Sciences, Tallinn, Estonia) for size exclusion chromatography (SEC). Subsequent 500 µl fractions were collected with αMEM according to the elution protocol recommended by the manufacturer.

The third method for EV/exosome preparation is a combination of the ultracentrifugation method and the SEC method. Accordingly, the conditioned serumfree medium from the taxol-treated MSC was centrifuged (360 g/10 min), then (2,000 g/10 min), and afterwards (10,000 g/30 min) to remove cells, dead cells and debris, respectively. Thereafter, the SEC method was applied by a 100-fold concentration of the supernatant via the Amicon® Ultra-15 centrifugal filter device (Millipore/Merck/Sigma-Aldrich Chemie GmbH) and subsequent separation with the SEC column. Two fractions containing the maximal number of EVs/exosomes as analysed by NTA (see below) were pooled and ultracentrifuged (100,000 g/70 min). The EVs/exosomes derived from the different preparation methods were stored at -80 °C in their present conditions and dilutions.

While some characterization for exosomal properties was performed according to the recently updated MISEV (minimal information for studies of extracellular vesicles) 2018 standards [42], the obtained vesicles were termed exosomes in this manuscript. Protein aliquots of the corresponding exosomes were quantified using the colorimetric BCA-assay (Thermo Scientific, Schwerte, Germany). The precipitated cell-derived exosomes were resuspended in 50 µl PBS and stored at -80 °C.

### Characterization of exosome preparations by Nanoparticle Tracking Analysis (NTA)

Aliquots of the different exosome preparations and SEC elution fractions in either PBS or αMEM were analyzed by nanoparticle-tracking-analysis (NTA) for vesicle concentration and size distribution using the ZetaView PMX120 NTA (Particle Metrix GmbH, Meerbusch, Germany) with an embedded 40 mW laser at 488 nm and a CMOS camera as extensively outlined previously [37].

### Immunoblot analysis of exosomes

Western blot analysis was performed as described elsewhere [11]. Briefly, exosome protein content was determined by the colorimetric BCA-assay (Perbio Science Deutschland, Bonn, Germany) and 20 µg protein aliquots of EV/exosome preparations from all untreated MSC controls and taxol-treated MSC cultures, respectively, were separated on a 10% SDS polyacrylamide gel and transferred to a nitrocellulose membrane (GE Healthcare Lifescience, Freiburg, Germany) after semi-dry blotting (Peqlab Biotechnology GmbH, Erlangen, Germany) at 1.5 mA/cm^2^ for 1 h. The blots were blocked with 5% milk (Marvel Freepost Premier Foods, Dublin, UK) in TBS-T buffer containing 0.1% sodium azide and incubated with a 1:500 dilution of the mouse monoclonal CD9 antibody (clone Ts9) (Invitrogen/ThermoFisher Scientific GmbH, Karlsruhe, Germany); a 1:300 dilution of the mouse monoclonal CD63 antibody (clone Ts63) (Invitrogen/ThermoFisher Scientific); a 1:300 dilution of the mouse monoclonal CD81 antibody (clone M38) (Invitrogen//ThermoFisher Scientific); a 1:1000 dilution of the rabbit monoclonal TSG101 antibody (clone EPR7130(B)) (abcam, Cambridge, UK).

Blocked immunoblots of cell lysates were incubated with a 1:1000 dilution of the rabbit monoclonal SDF-1 antibody (clone D32F9) (Cell Signaling Technology, Danvers, MA, USA), a 1:1000 dilution of the rabbit monoclonal CXCR4 antibody (clone EPUMBR3) (abcam); a 1:1000 dilution of the rabbit monoclonal CXCR7 antibody (clone SN65-09) (Invitrogen/ThermoFisher Scientific); a 1:200 dilution of the mouse monoclonal GAPDH antibody (clone 6C5) (Santa Cruz Biotechnology Inc., Heidelberg, Germany) After washing and incubation with a 1:5000 dilution of a corresponding HRP-conjugated sheep anti-mouse or donkey anti-rabbit secondary antibody (Cytiva Europe GmbH, Freiburg i.B., Germany), the membranes were washed again with TBS/Tween-20 and developed with the ECL reagent using the WesternBright Quantum HRP substrate (Advansta Inc., San Jose, CA). Visualization was performed by autoradiography using the ECL Hyperfilm (Cytiva Europe GmbH).

### Cytotoxicity measurements of exosomes by fluoroscan assay

Control and taxol-loaded exosomes from the different MSC cultures were dissolved in appropriate cell growth medium and different dilutions were incubated with in vitro cultures of different cancer cell populations. The proliferative capacity was evaluated by fluorescence measurement using the fluoroscan assay as previously described [43]. Briefly, 1000 cells/well of A549^GFP^, SK-OV-3^GFP^, HT-29^GFP^, CCF-STTG1^GFP^, and MDA-MB-231^GFP^ were seeded with standard culture medium (100 μl/well) in flat bottom 96- well plates (Nunc/ThermoFischer Scientific, Roskilde, Denmark) and incubated overnight to allow attachment. Thereafter, 100 µl of culture medium was added to the cells as a control and in further wells 100 µl of culture medium containing taxol substance and taxol-loaded exosomes were added to the cells, respectively. Following incubation for 72 h or 168 h, respectively, the medium was removed and the cells were lysed with 5% (w/v) SDS. Afterwards, the fluorescence intensities of GFP in the cell homogenate which corresponded to the appropriate cell number of cancer cells was measured at excitation 485 nm / emission 520 nm using the Fluoroscan Ascent Fl (ThermoFisher Scientific).

Partial neutralization of taxol-loaded exosomes-mediated cytotoxicity was performed by addition of 5 µg, 500 ng, or 50 ng, respectively, of a neutralizing mouse monoclonal SDF-1 antibody (clone #79,014) (R&D systems/BioTechne, Minneapolis, MN, USA) to the MSC-derived taxol-loaded exosomes. In a corresponding control experiment 5 µg of a mouse monoclonal IgG1 antibody (R&D systems/BioTechne, Wiesbaden, Germany) were added to the MSC taxol exosomes. Thereafter, 100 µl of the taxol exosomes/SDF-1 neutralizing antibody mixture or 100 µl of the taxol exosomes/IgG1 control antibody mixture was incubated with 100 µl of A549^GFP^ cancer cells for 72 h and cytotoxic effects were measured in a fluoroscan assay as described above. Prior to use the lyophilized SDF-1-neutralizing antibody was reconstituted in sterile PBS at a concentration of 0.5 mg/ml. The SDF-1 neutralizing capacity of this antibody is limited to a maximum of about 50% of chemotactic effects according to the manufacturer’s recommendation.

### Isolation and analysis of miRs from MSC-derived exosomes

High yield total RNA was isolated from MSC-derived EV/exosome preparations according to previous work [44] using the EVeryRNA™ EV RNA purification kit (#Every100B-1, System Biosciences (SBI), Palo Alto, CA, USA). Quantification of microRNAs in these exosome RNA preparations was performed with the Quant-iT™ microRNA Assay Kit (#Invitrogen Q32882, Invitrogen/ThermoFisher Scientific). The assay for these small RNAs (~ 20 bp) was used for initial sample concentrations ranging from 50 pg/μL to 100 ng/μL by providing a detection range of 1 ng to 100 ng of RNA according to the manufacturer’s recommendations.

Quantification of miRNA expression levels was estimated by transcript per million (TPM) as described [45]: Normalized expression = mapped readcount/total reads*1,000,000. Differential expression analysis of two samples was performed using the DEGseq R package. *P*-value was adjusted using Package ‘qvalue’ [46]. qvalue < 0.01 and |log2(fold-change)|> 1 was set as the significantly differential expression by default.

Gene Ontology (GO) enrichment analysis was used on the differentially expressed miRNAs. GOseq-based Wallenius non-central hyper-geometric distribution [47] which could adjust for gene length bias, was implemented for GO enrichment analysis.

### Effects of miRs on different *cancer* cell lines

Thirteen different miRs were purchased from Hycultec GmbH, Beutelsbach, Germany. These include: l hsa-miR-132-3p; hsa-miR-132-5p; hsa-miR-136-3p; hsa-miR-193-3p; hsa-miR-199-5p; hsa-miR-210-3p; hsa-miR-212-3p; hsa-miR-212-5p; hsa-miR-328-3p; hsa-miR-431-3p; hsa-miR-766-3p; hsa-miR-454-3p; hsa-miR-454-5p. Lipofectamine RNAiMAX reagent was obtained from Gibco/Life Technologies GmbH, Darmstadt, Germany.

For transfection, 1000 cells/well of A549^GFP^, SK-OV-3^GFP^, HT-29^GFP^, CCF-STTG1^GFP^, and MDA-MB-231^GFP^ were incubated overnight with corresponding culture medium (100 μl/well) without antibiotics to allow attachment in flat bottom 96- well plates (Nunc/ThermoFischer Scientific), respectively. Thereafter, 10 µl of transfection reagent containing 0.3 µl lipofectamine and 5 pmol miR in Optimem medium (Gibco/Life Technologies GmbH) was added to the 1000 cells/well for 6 h according to the manufacturer’s protocol. Following transfection, a medium exchange was performed with corresponding culture medium (200 μl/well) for further incubation of the cells. After a 72 h or 168 h culture, respectively, the medium was removed and the cells were lysed with 5% (w/v) SDS and the GFP fluorescence intensities in the cell homogenate were measured at excitation 485 nm / emission 520 nm using the Fluoroscan Ascent Fl (ThermoFisher Scientific).

### Detection and quantification of SDF-1 in MSC-derived exosomes by enzyme-linked immunosorbent assay (ELISA)

Protein aliquots (40 µg respectively) of control exosomes and corresponding taxol-loaded exosomes from 4 different individual MSC (MSC241111 P4; MSC280416 P6; MSC180314 P2; MSC270815 P4) were subjected to a sandwich enzyme-linked immunosorbent assay (ELISA) as previously described [48]. For the ELISA a SDF-1 kit (antibodies-online GmbH, Aachen, Germany) was used according to the manufacturer’s recommendations. After termination of the ELISA reactions by the addition of stopping solution, the absorbance of each well was measured with the Fluoroscan Ascent Fl (ThermoFisher Scientific) using a 450‐nm wavelength filter.

### Statistical analysis

Assays were performed by three independent experiments. Data represent the mean ± s.d. (*n* = 3). Significance of the data was calculated by unpaired two-sided student’s t-test. Significance of the symbols indicate: * = *p* < 0.05; ** = *p* < 0.01; *** = *p* < 0.005; **** = *p* < 0.001.

## Results

The transmembrane G-protein–coupled receptor CXCR4 is involved in numerous functions including lymphopoiesis, hematopoietic stem cell homing and engraftment, cell survival enhancement, and migratory activities among others. Thereby, CXCR4 can interact with its canonical ligand SDF-1. CXCR4 also plays a major role in a pathophysiological environment such as promoting tumor growth, e.g. by support of cancer cell migration and the formation of distant metastasis [49]. Accordingly, abundant CXCR4 expression is detectable on human breast cancer cells including MDA-MB-231 in contrast to normal human mammary epithelial cells [50]. In this context, various cancer cells demonstrate a significant expression of functional CXCR4 on the cell surface including A549 lung cancer [51], SK-OV-3 ovarian cancer [52], brain tumors like CCF-STTG1 astrocytoma [53], HT-29 colon adenocarcinoma [54], and much more tumor entities.

Moreover, another seven transmembrane-spanning receptor CXCR7 (= atypical chemokine receptor-3 (ACKR3)) represents affinity to SDF-1 binding [55] suggesting that both, CXCR4 and CXCR7 can relay intracellular signals following stimulation by SDF-1.

### Characterization of MSC-derived exosomes

In extension of previous work [37], a comparative examination of EV/exosome production was performed in four individual primary human MSC cultures (MSC241111, MSC280416, MSC180314, and MSC270815) and in the human MSC544 cell line.

**Fig. 1:**
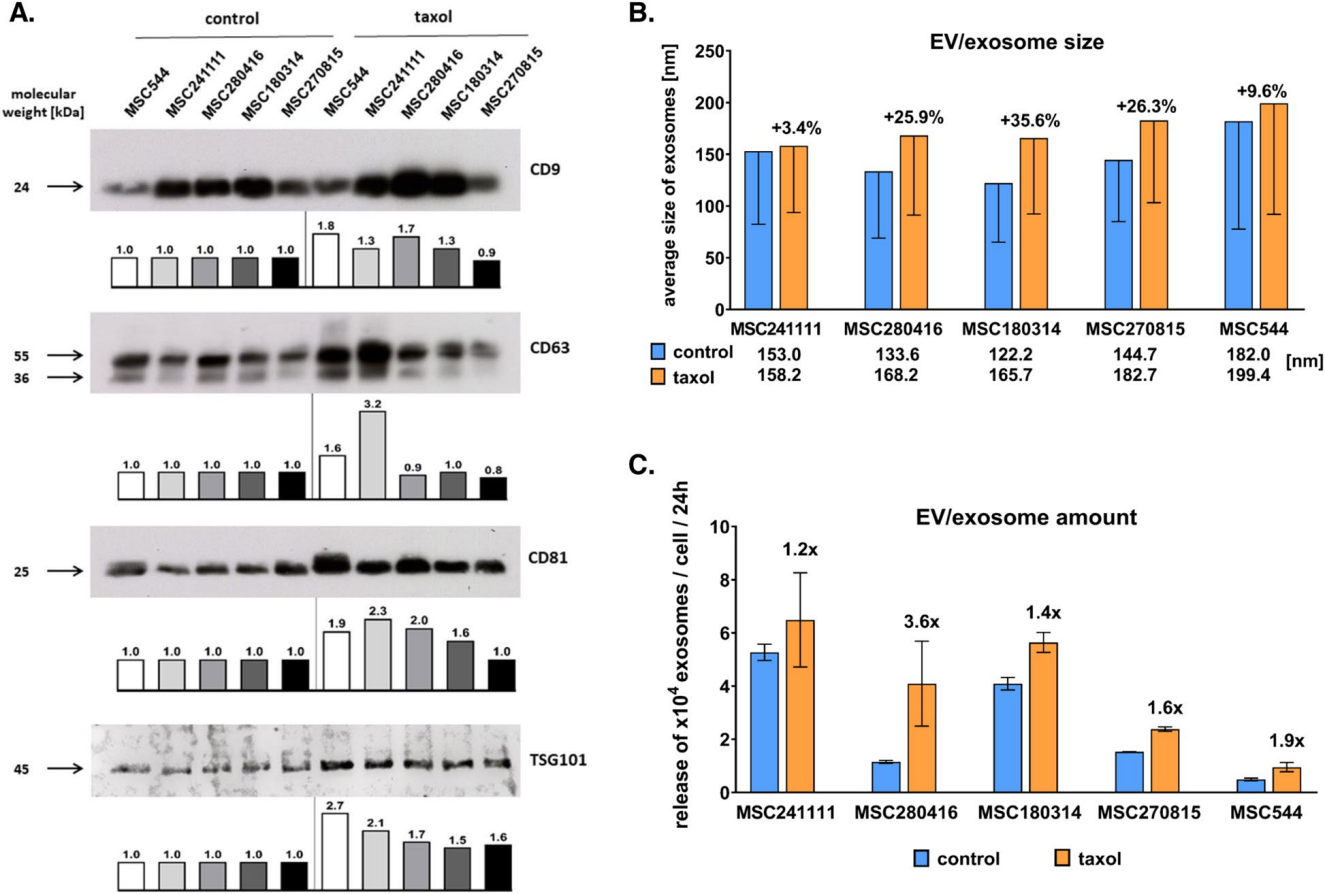
Characterization of MSC-derived EVs/exosomes. Western blots were performed with 20 µg protein/lane of untreated control exosomes (control) and corresponding taxol-loaded exosomes (taxol) from different individual MSC. The intensities were quantified using image J densitometry analysis by comparison of the controls (= 1) to their corresponding taxol treatment (**A**). (Original Western blot data are documented in suppl. Fig. S2). The different exosome preparations from the various MSC populations were analyzed by nano-tracking-analysis (NTA). Average diameters in [nm] are listed below the graph and differences in size were calculated as percentage between untreated control exosomes and their corresponding taxol-loaded exosomes (**B**). Quantification of the amount of exosome production was also determined by NTA and calculated as released exosomes per cell per 24 h. The fold differences (increase) of taxol-loaded exosomes as compared to the corresponding untreated control exosomes are indicated above the taxol bars (**C**). Data represent the mean ± s.d. (*n* = 3)

Following incubation of MSC control and taxol-treated MSC with serumfree medium for 24 h, EV/exosome production was isolated by differential ultracentrifugation. The vesicles were analyzed by transmission electron microscopy as described previously [56] (suppl. Fig. S1) and quantified by immunoblots (Fig. 1A) and by nano-tracking-analysis (NTA) (Fig. 1B, C). Expression of exosome-specific proteins like the tetraspanins CD9, CD63, and CD81, and TSG101 as an integral protein of the ESCRT (endosomal sorting complex required for transport) machinery were detectable in all MSC-derived exosome preparations (Fig. 1A; suppl. Fig. S2). Quantification by densitometry analysis revealed mostly an increased expression in the taxol-loaded exosomes as indicated by the intensity bars and relative induction numbers when compared to the corresponding control exosome expression levels (Fig. 1A).

While the four primary human MSC cultures (MSC241111, MSC280416, MSC180314, and MSC270815) produced an average amount of 3.0 × 10^4^ ± 1.4 × 10^3^ EVs/exosomes per cell within 24 h the human cell line MSC544 released 1.96 × 10^3^ ± 53.1 particles revealing an about tenfold reduced capacity for EVs/exosomes production as compared to the primary cultures. Accordingly, further therapeutic testing was focused on the more efficient MSC primary cultures.

Of interest, EVs/exosomes from all taxol-stimulated MSC demonstrated an increase in size (volume) between 3.4% and 35.6% as compared to the corresponding control MSC (Fig. 1B).

Moreover, all four primary human MSC cultures and the MSC544 cell line exhibited a markedly elevated amount of EVs/exosomes per cell following taxol treatment (ranging between 1.2-fold and 3.6-fold) as compared to the untreated MSC populations (Fig. 1C).

To prove effective washing of MSC before incubation of the cells with serumfree medium for EV/exosome production, EVs were isolated in a control experiment from the third washing step. NTA revealed a 444 ± 109 (*n* = 4) times reduced amount of EVs/exosomes indicating no significant effect of these remaining particles for the culture.

### Therapeutic effects of taxol-loaded MSC-derived exosomes

**Fig. 2:**
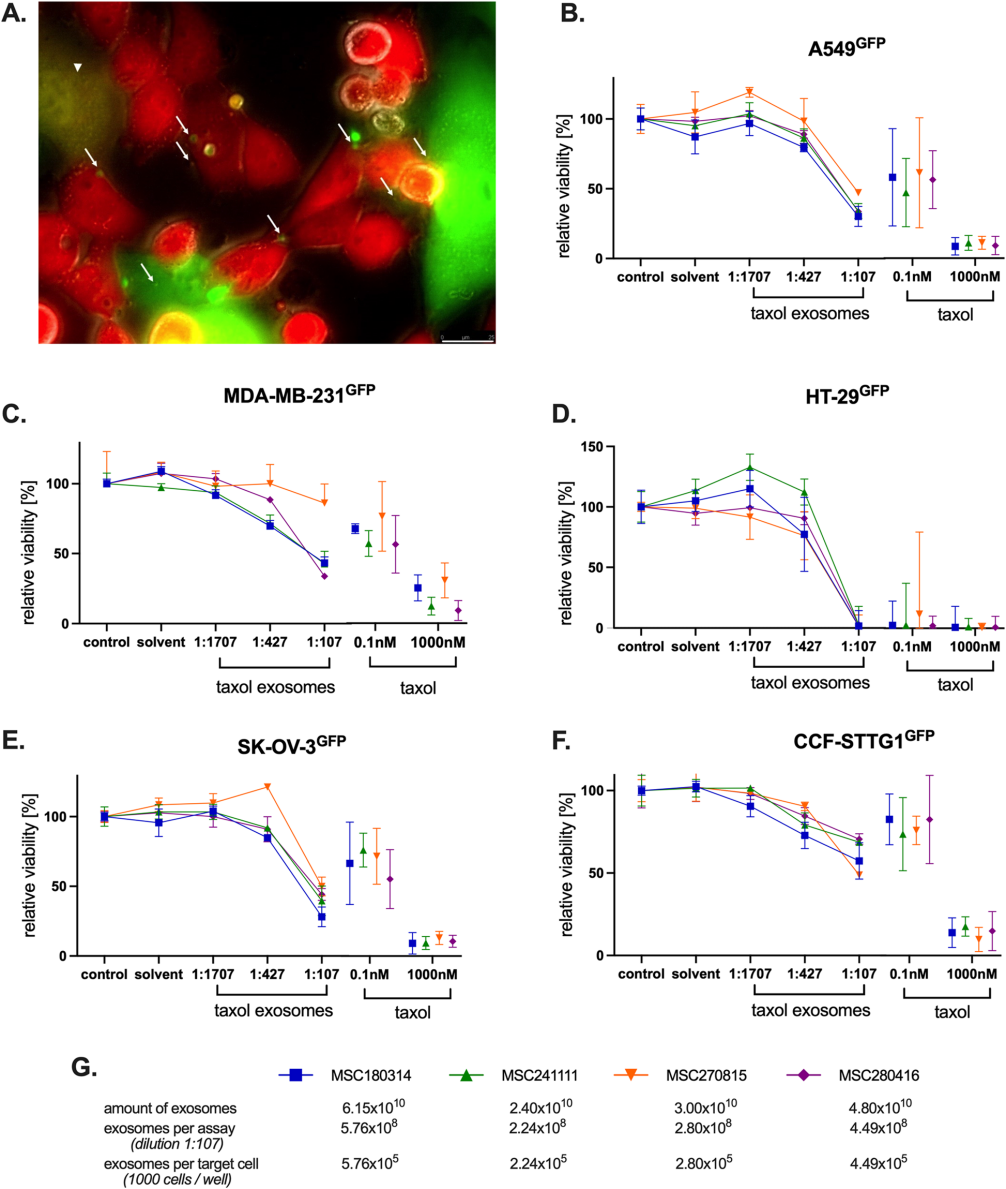
Cytotoxic effects of various MSC-derived taxol-loaded EVs/exosomes on different cancer cell types. Interaction of MDA-MB-231^cherry^ human breast cancer cells with MSC060616^GFP^ was documented after a 72 h co-culture with an initially cell ratio 50:50. Uptake of MSC^GFP^-released GFP-labeled EVs/exosomes by MDA-MB-231^cherry^ cherry cells are indicated by white arrows. A breast cancer hybrid cell after spontaneous fusion of MDA-MB-231^cherry^ with MSC060616^GFP^ is documented by the white arrowhead (bar = 25 µm) (**A**). Taxol-loaded exosomes isolated from four different individual MSC (MSC241111 P4; MSC280416 P6; MSC180314 P2; MSC270815 P4) as indicated in the legends were incubated with A549^GFP^ lung carcinoma cells for 72 h (**B**), with human MDA-MB-231^GFP^ breast carcinoma cells for 72 h (**C**), with slow-growing human HT-29^GFP^ colon adenocarcinoma cells for 168 h (**D**), with human SK-OV-3^GFP^ ovarian cancer cells for 72 h (**E**), and with slow-growing human CCF-STTG1^GFP^ astrocytoma cells for 168 h (**F**). The different GFP-labeled tumor cell lines were treated with appropriate amounts of taxol-loaded exosomes as indicated in the figures. The taxol-loaded exosomes were applied to the tumor cell lines in different dilutions (1:107; 1:427; 1:1707) and the viability was evaluated by fluoroscan assay as compared to two different taxol concentrations (0.1 nM; 1000 nM), the taxol solvent (solvent), and cell culture medium (control) as controls. Data represent the mean ± s.d. (*n* = 3)

Interaction of MSC with cancer cells either by direct cell-to-cell communication or by indirect processes including the release of EVs/exosomes has been studied previously [57, 58]. Thus, green-fluorescing EVs/exosomes derived from MSC060616^GFP^ are incorporated during co-culture by cancer cells such as human MDA-MB-231^cherry^ breast cancer cells (Fig. 2A, white arrows). Of interest, the co-culture also demonstrated a double-labeled breast cancer hybrid cell after spontaneous fusion of MDA-MB-231^cherry^ with MSC060616^GFP^ (white arrowhead) as previously described [59].

In the course of cellular interactions, MSC-derived EV/exosome-containing cargo can be transferred to recipient cells by promoting functional changes [60]. Consequently, EVs/exosomes isolated from different individual taxol-treated MSC-derived were used as a vehicle for carrying the chemotherapeutic cargo to various human cancer cells. The cytotoxic effects of the taxol-loaded EVs/exosomes on the GFP-tagged cancer cells were quantified as relative viability in a fluoroscan assay (Fig. 2B to F). Fluorescence micrographs substantiated a cytotoxic effect of taxol rather than a reduced proliferation rate (suppl. Fig. S3).

In extension of previous studies with an exosome mixture of taxol-treated MSC [37], the present work discriminated distinct anti-tumor effects of taxol-loaded exosomes isolated from four individual MSC populations. According to altered proliferative capacities of different tumor entities, more rapidly growing cancer cells including the A549^GFP^ lung carcinoma, MDA-MB-231^GFP^ breast carcinoma, and SK-OV-3^GFP^ ovarian cancer cells were incubated for 72 h while the slower proliferating HT-29^GFP^ colon adenocarcinoma and CCF-STTG1^GFP^ astrocytoma cells were treated for 168 h, respectively.

Overall, EVs/exosome preparations of all individual taxol-treated MSC exhibited a concentration-dependent cytotoxicity after application to the various cancer cells. Obvious differences applied to taxol-loaded EVs/exosomes from MSC270816 demonstrating a lower cytotoxic effect on the high proliferative cancer cells (A549^GFP^, MDA-MB-231^GFP^, SK-OV-3^GFP^) as compared to the other MSC populations (Fig. 2B, C, E). Conversely, these taxol-loaded EVs/exosomes from MSC270816 displayed higher cytotoxic effects on the lower proliferating cancer cells HT-29^GFP^ and CCF-STTG1^GFP^ (Fig. 2D, F). Moreover, the different individual MSC-derived taxol-loaded EVs/exosomes exhibited a cytotoxicity compared to about 1 nM to 10 nM of taxol substance that is in well agreement with the mixture of taxol exosomes in previous studies [37].

Differences in the effectiveness of the EVs/exosomes among the individual MSC sources remained negligible for the highest amount applied (1:107) while between 2.24 × 10^5^ EVs/exosomes (from MSC241111) and 5.76 × 10^5^ EVs/exosomes (from MSC180314) were used per target cancer cell (Fig. 2G). To estimate the amount of taxol in these MSC-derived EVs/exosomes, extensive LC–MS/MS analyses in a previous work have demonstrated approximately 123 ± 0.7 nM taxol in the EV/exosome preparations [37]. Accordingly, a 1:107 dilution of the EV/exosome preparation in the present study represents an equivalent of about 1.15 nM taxol. These findings suggested certain homogeneity of taxol cargo in equivalent amounts of EVs/exosomes derived from MSC sources of different individuals further supporting a potentially allogeneic clinical use as cell-free therapeutics.

**Fig. 3:**
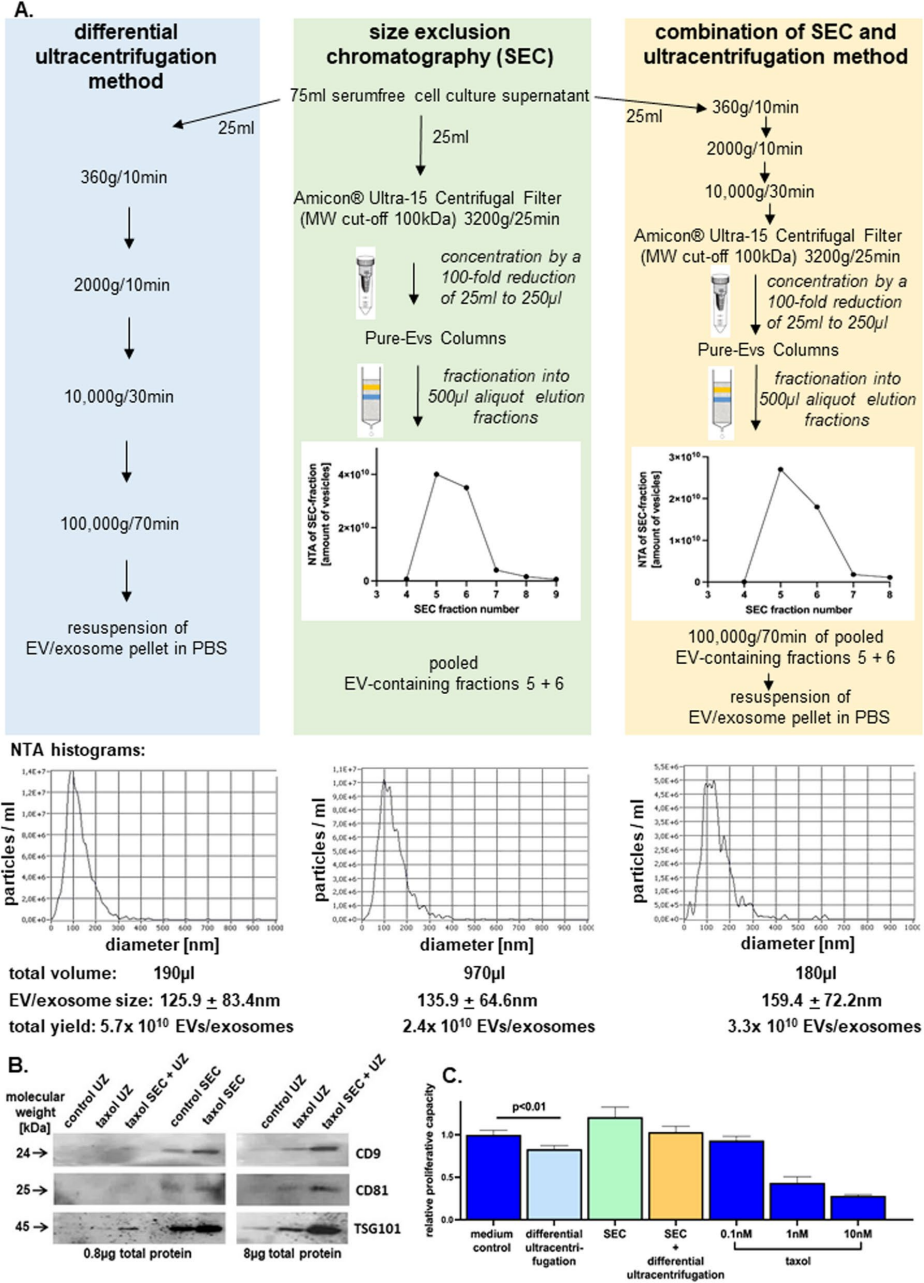
Comparison of EV isolation protocols. EV/exosome isolation of a 75 ml culture supernatant derived from 10 µM taxol-treated MSC after 24 h in serumfree medium was performed by the three different methods with 25 ml each as indicated. After size exclusion chromatography (SEC) the 500 µl fractions were analysed by NTA and reproducibly revealed the maximal EV-concentrations in fractions 5 and 6 which were pooled, respectively. Analyses of the NTA histograms revealed different yields from the three different methods applied (**A**). The EV/exosome isolation methods from untreated MSC (control) or taxol-treated MSC (taxol) by differential ultracentrifugation (UZ) and/or SEC were compared by Western blot analysis for expression of tetraspanins. After the removal of non-EV proteins and due to the eluate dilution after SEC, only 0.8 µg of total protein were loaded per lane (**B**, left panel). A more detailed comparison between EV/exosomes isolated by UZ from untreated MSC (control UZ), taxol-treated MSC (taxol UZ), and EV/exosomes isolated by a combination of SEC and UZ (taxol SEC + UZ) was performed by loading 8 µg of total protein per lane for the Western blot analysis of EV/exosome markers CD9, CD81, and TSG101 (**B**, right panel). For a direct comparison of chemotherapeutic effectiveness among the three different preparation methods the number of vesicles was normalized in each assay. Accordingly, 4.8 × 10^9^ EVs/exosomes of either preparation method were incubated with A549^GFP^ cells (1000 cells/well) in a microtiter plate for 72 h, respectively. Untreated A549^GFP^ cells served as a control (medium control) and the different concentrations of taxol substance (0.1 nM; 1 nM; and 10 nM) were used as a reference for cytotoxicity (**C**). Data represent the mean ± s.d. of experiments with three replicates

For potential clinical use, appropriate EV isolation methods require an optimized yield and chemotherapeutic effectiveness. Accordingly, alternative EV isolation methods were tested including size exclusion chromatography (SEC) or a combination of both, differential ultracentrifugation and SEC to putatively increase purity and efficiency as briefly outlined in Fig. 3A. Taxol-loaded EVs/exosomes derived from the same taxol-treated MSC280416 population were isolated in equal volumes of 25 ml by the ultracentrifugation method and compared to SEC-separated vesicles or a combination of these two methods. In contrast to a yield of 5.7 × 10^10^ EVs/exosomes with the differential ultracentrifugation method, the SEC revealed only 2.4 × 10^10^ EVs/exosomes and the combined method 3.3 × 10^10^ EVs/exosomes demonstrating an about 50% loss in yield with the alternative EV isolation methods (Fig. 3A). Western blot analyses of the different EV/exosome isolation methods revealed a predominant accumulation of the tetraspanins CD9 and CD81, and TSG101 in the SEC eluates after removal of non-EV proteins. Due to a low protein concentration in the diluted SEC samples (control SEC, taxol SEC) 0.8 µg of total protein were loaded per lane (Fig. 3B, left panel). A comparison of the more concentrated EV/exosome isolates also demonstrated an enhanced tetraspanin accumulation in the taxol EVs/exosomes and further enrichment of EV-markers in the SEC + UZ after removal of non-EV proteins (Fig. 3B, right panel). Effectiveness of equal numbers of the different EV/exosome preparation methods was tested by cytotoxic effects on the GFP-tagged A549 cells and relative viability was quantified in a fluoroscan assay. Thus, MSC-derived taxol-loaded exosomes isolated by the differential ultracentrifugation method revealed a significant cytotoxicity as compared to untreated A549^GFP^ cancer cells in control medium. In contrast, there was little if any growth-reducing effect detectable with the same amount of EVs/exosomes from SEC or a combination of both, differential ultracentrifugation and SEC (Fig. 3C). The loss of cytotoxicity in the taxol-EVs of the SEC method as compared to the combined SEC + differential ultracentrifugation method may be explained in part by the previous freezing of these EVs in a large volume. Whereas no optimal conditions for EV storage have been determined to date, these nanoparticles may undergo various degrees of change during cryo-preservation affecting their size, shape, content, and function [61, 62].

### MicroRNA (miR) characterization in MSC-derived exosomes

**Fig. 4:**
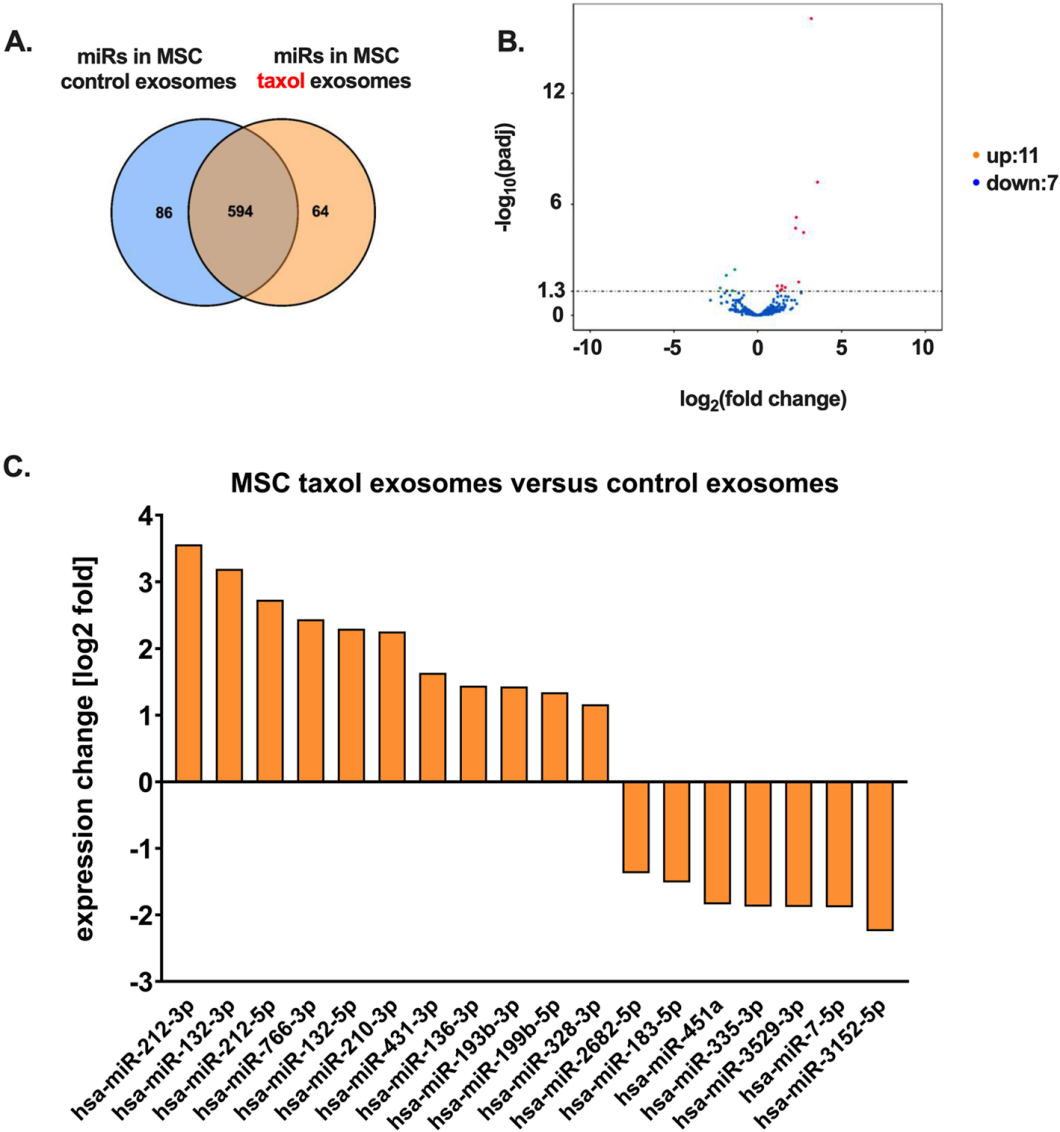
Characterization of miRs in MSC-derived control and taxol-loaded EVs/exosomes. The common micro RNA (miR) pattern obtained from isolated exosomes of four different MSC (MSC241111 P4; MSC280416 P6; MSC180314 P2; MSC270815 P4) as MSC control exosomes was compared to a common pattern of appropriate miRs obtained from taxol-loaded exosomes of these four MSC. Beside unique miRs of MSC control exosomes (violet circle) and MSC taxol exosomes (yellow circle) the overlap of both circles displays the amount of common miRs expressed in MSC control and taxol exosomes (**A**). Further analysis of the common miRs expressed in MSC control and taxol exosomes was performed for differential expression of up- and down-regulation as displayed in a volcano blot (**B**). The 18 differentially expressed miRs from the volcano blot in (**B**) with > log2-fold changed expression levels were identified with correlated hybridizing hsa-antagomirs and displayed for up- and down-regulated miRs (**C**)

Analysis of the miR patterns in MSC-derived control and taxol exosomes was performed for 1246 miRs. For this, the expression levels of miRs obtained from isolated exosomes of four different individual MSC (MSC241111 P4; MSC280416 P6; MSC180314 P2; MSC270815 P4) were combined to a pattern of miRs in MSC control exosomes. Likewise, miRs of the corresponding four taxol-treated MSC were combined to represent a common pattern of MSC-derived taxol exosomes. A comparison of MSC-derived control and taxol exosome miR expression levels revealed 86 unique miRs in the control MSC while 64 miRs exclusively appeared in exosomes of the corresponding taxol-treated MSC. The majority of 594 miRs was detectable in exosomes of both, control and taxol-treated MSC (Fig. 4A). While unique miR expression can be related to individual properties of certain MSC further analysis was performed regarding the 594 commonly expressed miRs. Vulcano blot analysis revealed most miRs (576) at similar expression levels whilst 18 miRs appeared at significantly different levels with more than log2-fold changes. These 18 miRs included an up-regulation of 11 miRs and a down-modulation of 7 miRs in the MSC-derived taxol exosomes as compared to control exosomes (Fig. 4B). Characterization of these differentially expressed miRs demonstrated an up-regulation of various miRs with tumor-suppressive effects.

Thus, the most prominently expressed miRs in exosomes of taxol-treated MSC include miR-212-3p (Fig. 4C). This miR is involved in inhibition of angiogenesis in various kinds of tumors such as breast cancer [63] and functions as a tumor suppressor in different tumor entities. In particular, miR-212-3p is involved in the inhibition of different tumor types of the brain [64, 65], suppression of ovarian cancer progression [66], and inhibition of bladder cancer [67]. Likewise, the second largest miR expression is represented by miR-132-3p (Fig. 4C) that acts as a tumor suppressor in a variety of solid tumors [68]. Moreover, the significantly induced miR-212-5p displays protective effects and can inhibit malignant development in gliomas, nasopharyngal tumors, clear cell renal carcinoma, and suppresses epithelial-mesenchymal-transition in triple-negative breast cancer [69]. An increased expression of miR-766-3p can promote p53 accumulation and G2/M cell cycle arrest [70]. Although the precise role of miR-766-3p in tumor-promoting and tumor-inhibiting activities is less clear, strong evidences revealed that decreased miR-766-3p levels are associated with a poorer prognosis and clinical stage in renal cell carcinoma patients. In triple-negative breast cancer upregulation of miR-766 dramatically lowers the formation of lung metastases and the studies indicated predominant effects on metastatic spreading than on primary tumor formation [71]. Other up-regulated miRs in MSC-derived taxol exosomes including miR-431-3p, miR-136-3p, miR-193-3b, miR-199-5b, and miR-328-3p can promote tumor growth in some cases but also inhibit tumor growth in various different tumors. Finally, miR-210-3p expression also inhibits growth and metastatic spreading of bladder cancer [72].

**Fig. 5:**
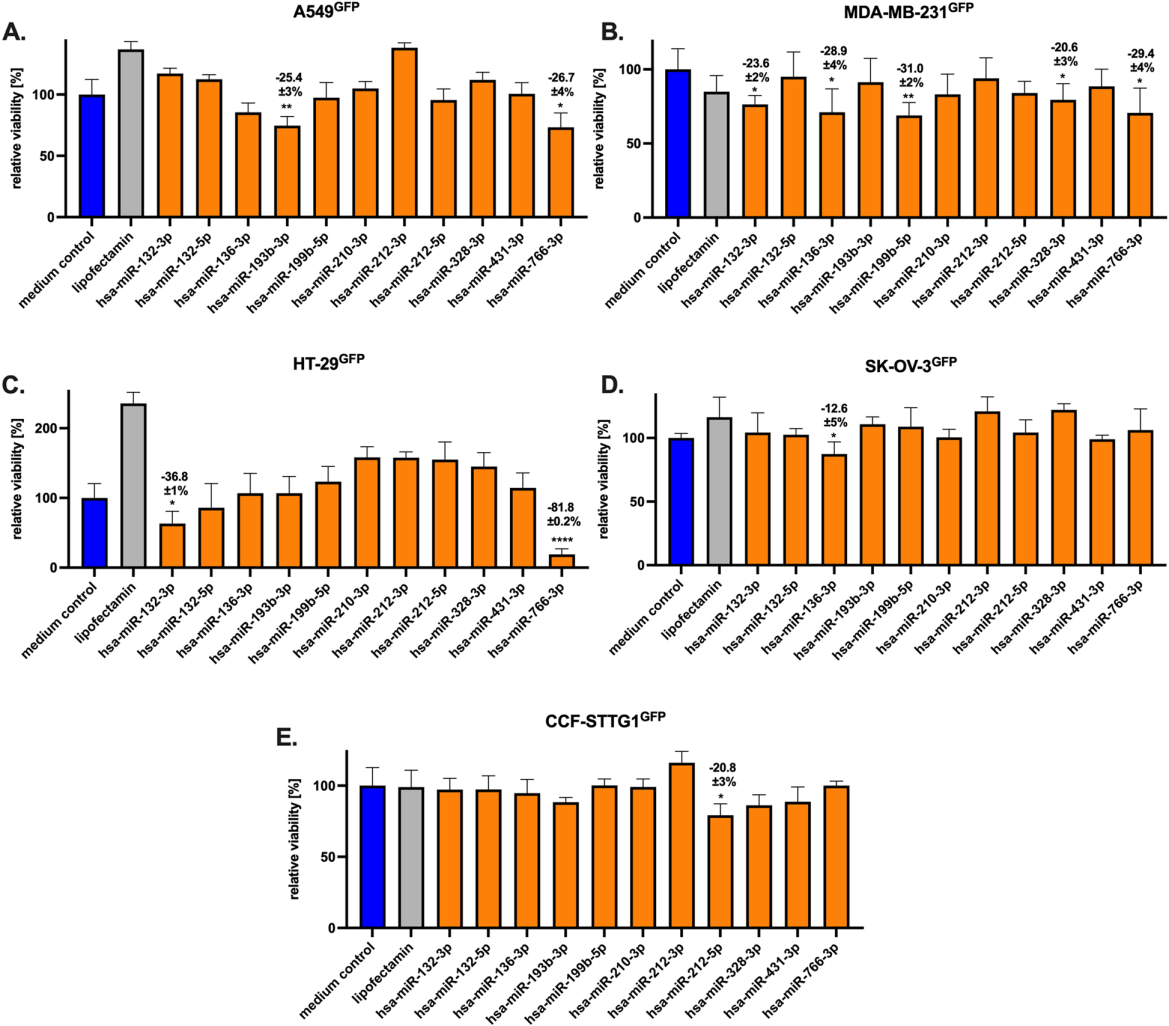
The role of miRs on the viability of different cancer cell lines. Following transfection with the indicated miRs, different GFP-labeled human cancer cell lines including A549^GFP^ lung carcinoma, MDA-MB-231^GFP^ breast carcinoma, and SK-OV-3^GFP^ ovarian cancer cells were incubated for 72 h while slow-growing HT-29^GFP^ colon adenocarcinoma and CCF-STTG1^GFP^ astrocytoma cells were cultured for 168 h. Thereafter, the viability was evaluated by fluoroscan assay as compared to lipofectamine alone and the corresponding cell culture medium (medium control). Data were normalized to the medium control and represent the mean ± s.d. of three independent experiments (*n* = 3)

To substantiate potential growth-reducing effects of the taxol-induced miRs in MSC EVs/exosomes, the different GFP-labeled cancer cell lines were transfected with each of the 11 miRs and the cell viability was analyzed by fluoroscan assay after 72 h (Fig. 5). While lipofectamine transfection caused both, stimulatory or inhibitory effects depending on the cell line, a lipofectamine concentration-dependency did not reveal significant differences (suppl. Fig. S4) and therefore, 0.3 µl lipofectamine/1000 cells was used throughout according to the manufacturers recommendation. The viability of miR-transfected cells as compared to the medium control displayed different effects among the various cell lines. A significant growth reduction in A549^GFP^ cells was observed with miR-193b-3p (25.4 ± 3%) and miR-766-3p (26.7 ± 4%) (Fig. 5A). In MDA-MB-231^GFP^ cells a variety of miRs reduced the population growth including miR-132-3p (23.6 ± 2%), miR-136-3p (28.9 ± 4%), miR-199-3b (31.0 ± 2%), miR-328-3p (20.6 ± 3%), and miR-766-3p (29.4 ± 4%) (Fig. 5B). A pronounced reducing effect of miR-766-3p was also observed in HT-29 cells with 81.8 ± 0.2% together with miR-132-3p (36.8 ± 1%) (Fig. 5C). Other miRs such as miR-136-3p reduced the growth of SK-OV-3^GFP^ cells by 12.6 ± 5% (Fig. 5D) and miR-212-5p diminished the growth of CCF-STTG1^GFP^ cells by 20.8 ± 3% (Fig. 5E). Together, these findings demonstrate growth-reducing effects of different miRs on distinct cancer cell lines suggesting potentially selective miR effects on certain tumor entities.

With respect to down-modulated miRs in exosomes of taxol-exposed MSC compared to control MSC the most reduced miR-3152-5p is involved in targeting the Ser/Thr kinase HipK2. Further reduced miRs such as miR-7-5p regulate chemoresistance in colorectal cancers and therefore, are also involved in the inhibition of cancer stem cell formation in colorectal tumors [73]. Tumor-associated functions of further down-modulated miRs in taxol exosomes are less clear. Of interest, miR-335-3p can serve as a tumor biomarker [74]. Particularly in MSC, miR-335 contributes to proliferation, migration and differentiation [75] and other work has suggested that the expression levels of miR-335 together with miR-126 can determine the activation status of MSC [76].

Alltogether, these data demonstrated that up-regulation of various miRs in EVs/exosomes of taxol-treated MSC can be associated predominantly with tumor-suppressive effects whilst down-modulated miRs can exhibit multiple effects. Detailed analyses of all investigated miRs in EVs/exosomes of taxol-treated MSC as compared to the EVs/exosomes miR pattern of the corresponding MSC control populations are documented in suppl. Fig. S5. Moreover, gene ontology analysis was performed with the miR data from MSC-derived taxol versus control EVs/exosomes. Differences in the miR pattern of taxol EVs/exosomes versus control EVs/exosomes was discriminated according to biological processes, cellular components, and molecular function. Accordingly, EVs/exosomes after taxol treatment of all four investigated MSC predominantly affected enhanced Ras-/Rho-GTP signaling and reorganization of actin-associated cytoskeletal structures (suppl. Fig. S6).

### SDF-1 detection in MSC-derived exosomes

**Fig. 6:**
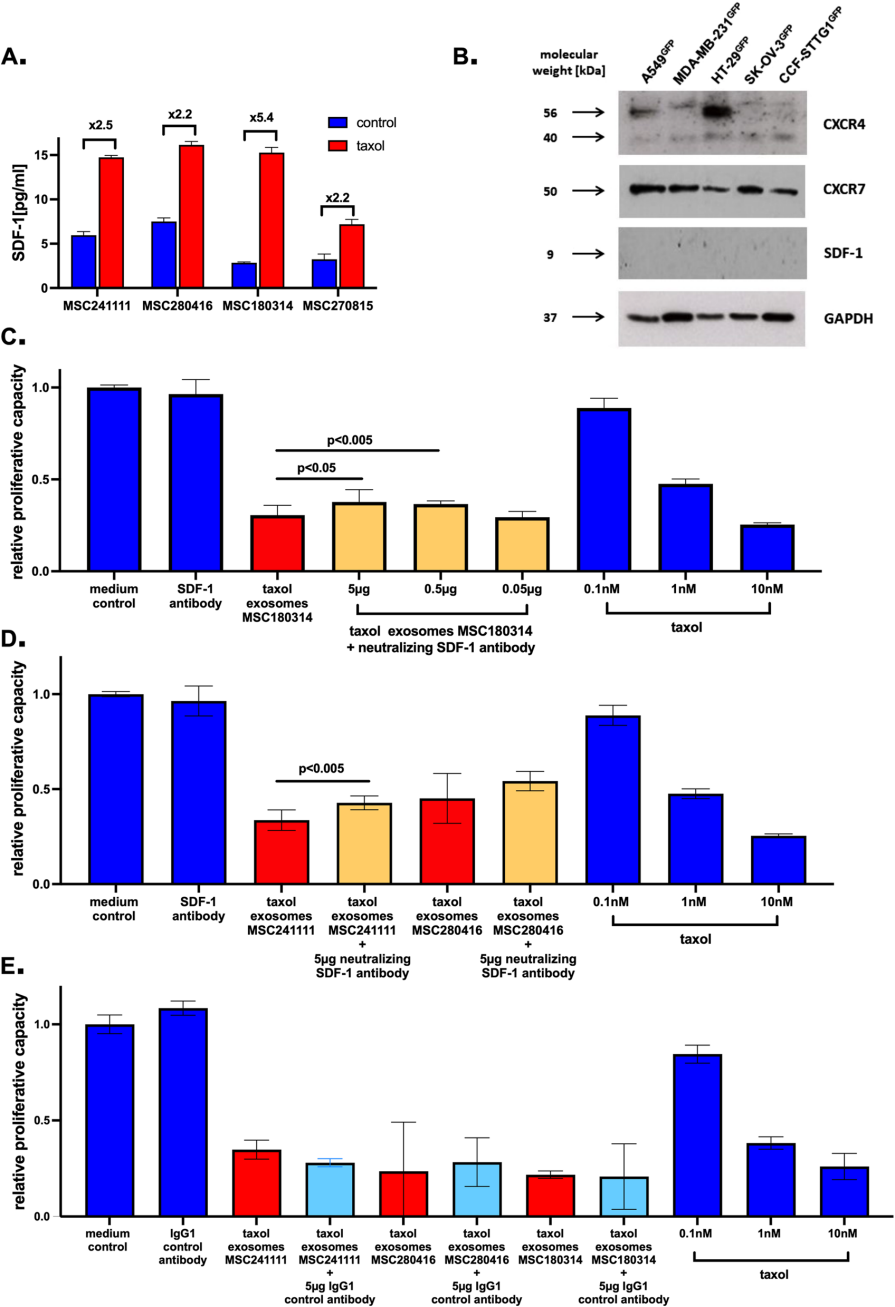
Role of SDF-1 on cytotoxic effects of MSC-derived taxol-loaded EVs/exosomes. Following exosomes isolation of four different individual MSC (MSC241111 P4; MSC280416 P6; MSC180314 P2; MSC270815 P4) and of the corresponding taxol-loaded MSC, equal aliquots of 40 µg exosome protein were subjected to an ELISA for quantification of SDF-1. Data represent the mean ± s.d. (*n* = 3) whereby the numbers above brackets of the bars were calculated as fold induction of SDF-1 in taxol exosomes when compared to sdf-1 level in control exosomes (**A**). Western blot analysis of cancer cell lines representing five different tumor entities was performed for the expression of the specific SDF-1 receptors CXCR4 and CXCR7. The ligand SDF-1 remained undetectable and expression of GAPDH served as a control (**B**). Different concentrations of a neutralizing SDF-1 antibody were mixed with taxol-loaded exosomes (1:107) from MSC180314 and incubated together with A549^GFP^ lung carcinoma cells. After 72 h of co-culture the cytotoxicity was evaluated by fluoroscan assay similar to the method applied in Fig. 2B (see above) (**C**). Taxol-loaded exosomes (1:107) from MSC241111 and MSC280416 were mixed with 5 µg of SDF-1 neutralizing antibody, respectively, (**D**) or with 5 µg of an IgG1 control antibody, respectively, (**E**) and incubated together with A549^GFP^ lung carcinoma cells for 72 h. Thereafter, the cytotoxicity was evaluated by fluoroscan assay. Data represent the mean ± s.d. (*n* = 3)

Taxol treatment of different MSC populations was associated with markedly increased SDF-1 production as analysed by a SDF-1 ELISA. Quantification of SDF-1 revealed detectable amounts in all exosome samples whereby the concentration of SDF-1 protein was significantly enhanced by 2.2-fold to 5.4-fold in all MSC-derived taxol-loaded exosomes when compared to MSC control exosomes (Fig. 6A).

While previous work in a pancreatic ductal adenocarcinoma model has demonstrated that miR-454 binds to the 3′-untranslated region of SDF-1 mRNAs and inhibits SDF-1 protein translation [77] it was suggested that reduced miR-454 contributes to enhanced SDF-1 exppression. According to a reduced miR-454 expression in MSC populations after taxol treatment we addressed the question as to whether the taxol-mediated increase in SDF-1 production might be mediated via miR-454. Transfection of three different MSC populations with miR-454 and subsequent quantification of SDF-1 production by a corresponding ELISA, however, demonstrated little if any effects on the SDF-1 production of either MSC population (suppl. Fig. S7). These findings suggested that a certain sustained threshold of orchestrating miRs may promote cellular changes more profoundly rather than local short-term single miR signaling. Whereas SDF-1 represents the ligand for the chemokine receptors CXCR4 and CXCR7, Western blot analyses were performed to identify expression of these receptors on the different cancer cell lines representing five different tumor entities. Both, CXCR4 and CXCR7 demonstrated a prominent presence on all of the different cancer cells. In contrast, little if any signals were detectable for the expression of SDF-1 while GAPDH protein levels represented the control (Fig. 6B). These findings suggested that CXCR4 and CXCR7 on the different cancer cell types can be stimulated by exogenous SDF-1, e.g. from MSC-derived exosomes-associated SDF-1.

To test this hypothesis, we investigated a functional relevance of the increased exosomes-associated SDF-1 from taxol-treated MSC for interaction in a cancer cell line. For this approach, different amounts of a neutralizing SDF-1 antibody and MSC-derived taxol exosomes were incubated together with A549^GFP^ lung carcinoma cells for 72 h to examine cytotoxic effects.

Incubation of A549^GFP^ cells with 5 µg of neutralizing SDF-1 antibody alone displayed a similar viability like control medium whereas MSC-derived taxol-loaded exosomes markedly reduced the A549^GFP^ cell viability by 70% within 72 h. However, addition of 5 µg or 0.5 µg of neutralizing SDF-1 antibody to MSC-derived taxol exosomes demonstrated a significant concentration-dependent reduction of cell cytotoxicity, respectively. A further tenfold dilution of 0.05 µg neutralizing SDF-1 antibody displayed no significant differences in cytotoxicity as compared to MSC-derived taxol exosomes alone. Effects of taxol substance between 0.1 nM and 10 nM as a reference confirmed that the amount of applied MSC-derived taxol exosomes confer a comparable cytotoxicity like a 1 nM to 10 nM taxol treatment (Fig. 6C).

Based on the concentration-dependent effects of the neutralizing SDF-1 antibody in MSC180314, taxol-loaded exosomes of two other MSC primary cultures (MSC 241111 and MSC280416) were incubated with 5 µg of the neutralizing SDF-1 antibody and tested in A549^GFP^ cells in a parallel experimental setup. Both antibody incubations demonstrated a similar reduction of corresponding taxol exosome-mediated cytotoxicity suggesting that the neutralizing SDF-1 can partially abolish the taxol exosome effects disregard of the originating MSC population (Fig. 6D).

Moreover, taxol-loaded exosomes derived from all three MSC primary cultures (MSC 241111, MSC280416, and MSC180314) were incubated with 5 µg of an IgG1 control antibody, respectively and tested in A549^GFP^ cells in a similar experimental setup. In contrast to the results with the neutralizing SDF-1 antibody, there was little if any effect detectable on the taxol-loaded exosomes-mediated cytotoxicity by the IgG1 control antibody. Taxol substance between 0.1 nM and 10 nM was used again as a cytotoxicity control (Fig. 6E).

**Fig. 7:**
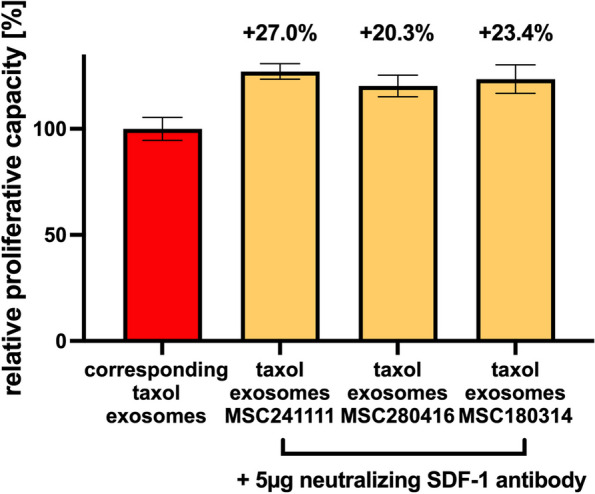
Rescue of MSC taxol exosomes-mediated cytotoxic effects by SDF-1 antibody Effects of 5 µg of the neutralizing SDF-1 antibody on taxol-loaded exosomes from the different MSC were calculated and compared to the effects of the corresponding taxol-loaded exosomes alone as a reference (= 100%). The percentages of increase, which are equivalent to an appropriately reduced cytotoxicity, are indicated above the bars. Each experiment represents the mean ± s.d. of three independent experiments

Quantification of the neutralizing SDF-1 antibody effects on MSC-derived taxol-loaded exosomes in A549 cells was performed by calculating the percentages of increase, which are equivalent to an appropriately reduced cellular cytotoxicity. Accordingly, the data revealed a reduction of the cytotoxicity between 20.3% and 27% when compared to the corresponding different MSC-derived taxol exosome (Fig. 7).

**Fig. 8:**
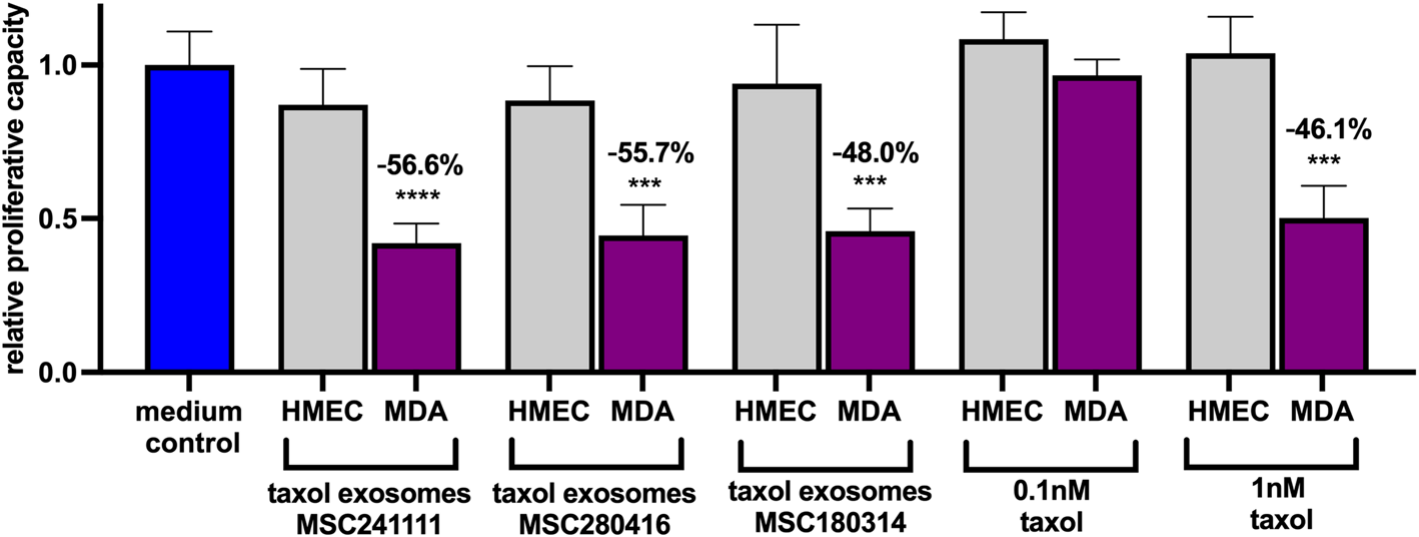
Effects of MSC taxol exosomes on breast cancer cells as compared to normal breast epithelial cells. GFP-labeled normal human mammary epithelial cells (HMEC) and MDA-MB-231^GFP^ breast cancer cells (MDA) were treated with taxol-loaded exosomes (diluted 1:107 similar to the treatment described in Fig. 2) from three different MSC populations (MSC241111; MSC280416; MSC180314) or with taxol (0.1 nM; 1 nM) for 72 h. Thereafter, the viability was measured by fluoroscan assay and the percentage of viable GFP-positive cells was calculated according to corresponding untreated populations (medium control) after 72 h. The numbers above the stars of significance indicate the percentage of reduced viability. Data represent the mean ± s.d. (*n* = 3)

Together, these findings suggested that SDF-1 in MSC-derived exosomes at least partially contributes to direct these nanovesicles towards CXCR4-/CXCR7-carrying cells. In addition, the significantly elevated SDF-1 expression in taxol-treated MSC-derived exosomes could enhance the specific binding to receptor-expressing cancer cells, which markedly increases a tropism towards tumors by a targeted delivery of the taxol-induced cargo. However, further yet unidentified components and mechanisms may also promote tumor tropism. Enhanced tropism of taxol to primary cultures of human breast cancer-derived epithelial cells (HBCEC) as compared to primary cultures of normal human mammary epithelial cells (HMEC) has been described previously [78]. Likewise, taxol-loaded exosomes induced cytotoxicity preferably in human MDA-MB-231^GFP^ breast cancer cultures rather than in normal HMEC^GFP^. In more detail, the amount of viable GFP-positive cells was significantly reduced by about 50% after treatment of the MDA-MB-231^GFP^ cancer cells with the different taxol-loaded exosomes or with 1 nM taxol. In contrast, there was little if any significant effect of either MSC-derived taxol-loaded exosomes or 1 nM taxol on normal HMEC (Fig. 8). These findings were also substantiated by a corresponding morphology of the cell cultures (suppl. Fig. S8) furthermore underscoring an enhanced tumor tropism mediated by MSC-derived taxol-loaded exosomes.

## Discussion

Solid tumors can develop as an organ-like structure whereby cancer cells constantly interact with multiple other cell types of the tumor microenvironment such as immune cells and MSC [79, 80]. Different types of interaction between cancer cells and MSC within the connective tumor tissues involve the exchange of trophic factors and small vesicles. These vesicles include EVs/exosomes that carry various regulatory biological compounds such as proteins and miRs for cellular communications with other cells. Depending on their activation status MSC express a distinct pattern of miRs that can be transported via EVs/exosomes to interact e,g, with cancer cells. Vice versa, several studies have shown an ectopic expression pattern of certain miRs in cancers such as miR-18a-5p in breast cancer [81], hepatocellular carcinoma [82], prostate cancer and other cancers [83]. Mutual exchange of large amounts of miR-associated EVs/exosomes thereby contribute to alter functionalities in the target cells.

A potential clinical use of MSC-derived EVs/exosomes is attractive as a cell-free system due to a reasonable biocompatibility and a reduced immunogenicity as compared to a cellular system. Moreover, the bi-layered lipid structure of exosomes protects the genetic cargo from degradation and can fuse with target cells to deliver the MSC-derived regenerative content. Various studies have demonstrated the tissue repair potential of MSC-derived EVs/exosomes in inflammation and diseases [23, 84].

Alternatively, MSC-derived EVs/exosomes can also be used as a vehicle for the transport of therapeutic compounds. Previous work has demonstrated that MSC can be treated with sub-lethal concentrations of taxol whereby this tumor-therapeutic substance is packed into multivesicular bodies and secreted as EVs/exosomes [37]. Accordingly, MSC-derived taxol-loaded EVs/exosomes can be isolated and successfully applied to a variety of different cancer cells.

Taxol-loading of MSC-derived exosomes following preceding taxol treatment of MSC, simultaneously altered the expression of certain miRs and proteins, which are concomitantly transmitted to the EVs/exosomes. Thus, exposure of MSC to taxol increased the expression of several tetraspanins in the EVs/exosomes. This enables an enhanced formation of tetraspanin-enriched microdomains that contribute to a functional crosstalk among signaling pathways of targeted cells. Moreover, the Ras-/Rho-GTP signaling pathways together with a reorganization of actin-associated cytoskeletal structures were affected after taxol stimulation of MSC. In addition, taxol treatment significantly elevated the transcripts of several miRs that are functionally associated predominantly with tumor-suppressive effects. Consequently, transmission of these properties to MSC-derived taxol-loaded EVs/exosomes already promote distinct anti-cancer issues alone disregard of the drug.

A major limitation for successful cargo delivery by EVs/exosomes remains the difficulty to precisely target the cell type or tumor tissue whilst limiting off-target biodistribution. In comparison to the application of an equivalent amount of taxol substance, however, taxol-loaded MSC-derived EVs/exosomes exhibit a more effective tumor targeting [37]. This increased anti-tumor efficiency is associated with the presence of distinct factors in taxol-loaded EVs/exosomes. One of these factors is represented by SDF-1, which can contribute to enhanced tropism towards tumors e.g. by interacting with the CXCR4/CXCR7 axes. While taxol treatment even enhanced the SDF-1 expression in MSC, the derived EV/exosome-associated SDF-1 may also contribute at least partially to the activation of the CXCR4 and/or CXCR7 receptors on target cells of several tumor populations.

However, the use of MSC-derived EVs/exosomes as a vehicle to deliver tumor-therapeutic compounds may not apply to all kinds of substances and therefore predominantly displays drug-specific properties. Nevertheless, the effects of taxol on the elevated transcripts of miRs with tumor-suppressive functionalities paralleled by the enhanced tumor tropism of increased SDF-1 expression potentiated these drug results. Moreover, a SDF-1-directed addressing of cancer cells contributes to a reduction of unwanted side effects. Accordingly, taxol-loaded MSC-derived EVs/exosomes provide a variety of functional properties for a successful and target-specific clinical application in a tumor-therapeutic approach.

## Conclusions

MSC-derived therapeutic EVs/exosomes with altered miR and protein patterns contribute to tissue selectivity and can alter certain functionalities in target cells. Thus, a “natural” production of taxol-loaded MSC-derived EVs/exosomes as a therapeutic carrier may display some advantages as compared to synthetic or other taxol-tagged nanoparticles.

While taxol exposure of MSC was associated with an increased SDF-1 expression in the derived exosomes, this suggested a selective tropism towards CXCR4-/CXCR7-carrying tumors. Simultaneously, taxol treatment of MSC was associated with increased expression of various anti-tumorigenic miRs present in the derived EVs/exosomes. Accordingly, these miRs in taxol-loaded EVs/exosomes can enhance therapeutic effects by ameliorating entry into cancer cells and targeting functionalities in the primary tumor and in their metastatic progenies. Consequently, SDF-1 in MSC-derived taxol exosomes contributes to a selective targeting of tumors and metastases in orchestration with the delivery of anti-tumorigenic miRs on top of the taxol cargo. Thus, MSC-derived drug loading with taxol represents a promising approach to develop a targeted drug delivery system (TDDS).

## Supplementary Information

Additional file 1. Supplementary Figure S1: A representative transmission electron micrograph of a MSC241111-derived exosome preparation by the ultracentrifugation method was performed as described previously for similar other MSC-derived exosomes [37, 56]. The vesicles were varying in size between 50nm to 200nm with rounded shape and a double-membrane. The content of MSC-secreted exosomes includes among others proteinous precipitates (indicated by arrows). Bar represents 300 nm.

Additional file 2. Supplementary Figure S2: Original Western blot data of MSC-derived EVs/exosomes.

Additional file 3. Supplementary Figure S3: Human MDA-MB-231^GFP^ breast carcinoma were treated with 1µM taxol for 72h, and the slower proliferating HT-29^GFP^ colon adenocarcinoma and CCF-STTG1^GFP^ astrocytoma cells were exposed to 1µM taxol for 168h, respectively. Documentation of the cells was performed using a fluorescence microscope (Olympus IX50). Desintegration of cells and cellular debris are indicated by white arrows. Bars represent 200µm.

Additional file 4 . Supplementary Figure S4: Lipofectamine concentration-dependency in different cancer cell lines.

Additional file 5. Supplementary Figure S5: Heatmap cluster analysis of miRs in MSC-derived EVs/exosomes and taxol-treated MSC-derived EVs/exosomes.

Additional file 6. Supplementary Figure S6: Gene ontology (GO) analysis was performed in differentially expressed miRs of four MSC-derived taxol-loaded exosome populations (MSC241111 P4; MSC280416 P6; MSC180314 P2; MSC270815 P4) as compared to the corresponding control EV/exosome miRs of the four MSC. Pathways affected by the differential miR expressions were discriminated by biological process (BP), cellular component (CC), and molecular function (MF), respectively.

Additional file 7 . Supplementary Figure S7: The four different MSC populations were incubated in culture medium (medium control), after transfection with 0.3µl lipofectamine (lipofectamine), after transfection with lipofectamine and a mixture of 2.5pmol miR-454-3p and 2.5pmol miR-454-5p (miR-454), and after stimulation with 10µM taxol (taxol). Following 24h of incubation the amount of released SDF-1 into the culture medium was quantified by an appropriate ELISA and calculated according to an equivalent amount of 1000 cells. Data represent the mean + s.d. of three independent experiments.

Additional file 8. Supplementary Figure S8: GFP-labeled normal human mammary epithelial cells (HMEC^GFP^) and human breast cancer cells (MDA-MB-231^GFP^) were treated with taxol-loaded exosomes isolated from three different human MSC populations (MSC241111, MSC280416, and MSC270815) in a 96-well microtiter plate for 72h, respectively. Incubation of the cells with 1nM taxol for 72h served as a control. Documentation of the cells was performed using a fluorescence microscope (Olympus IX50). Desintegration of cells and cellular debris are indicated by white arrows. Bars represent 200µm.

## Data Availability

No datasets were generated or analysed during the current study.

## Abbreviations

EVs: Extracellular vesicles
miR: MicroRNA
MSC: Mesenchymal stroma/stem-like cells
NTA: Nano-tracking-analysis
SDF-1: Stroma cell-derived factor 1
