# Supplementary figures and images for: Human mesenchymal stroma/stem-like cell-derived taxol-loaded EVs/exosomes transfer anti-tumor microRNA signatures and express enhanced SDF-1-mediated tumor tropism
Source: Cell Commun Signal. 2024 Oct 17;22:506. doi: 10.1186/s12964-024-01886-2 (PMC11488203; doi:10.1186/s12964-024-01886-2)

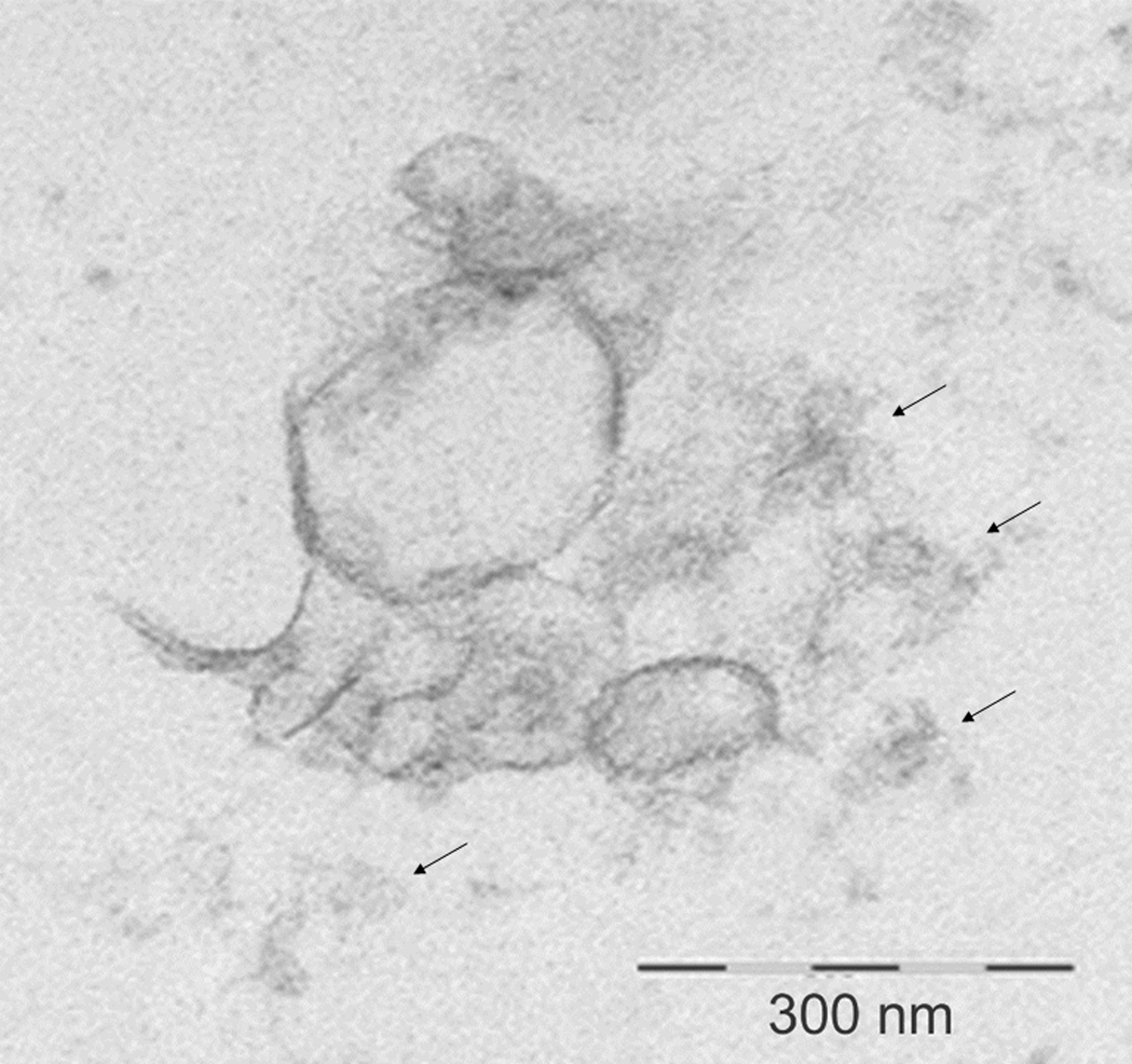

Supplement: Supplementary file 1 — Additional file 1. Supplementary Figure S1: A representative transmission electron micrograph of a MSC241111-derived exosome preparation by the ultracentrifugation method was performed as described previously for similar other MSC-derived exosomes [37, 56]. The vesicles were varying in size between 50nm to 200nm with rounded shape and a double-membrane. The content of MSC-secreted exosomes includes among others proteinous precipitates (indicated by arrows). Bar represents 300 nm. [file 12964_2024_1886_MOESM1_ESM.tif]

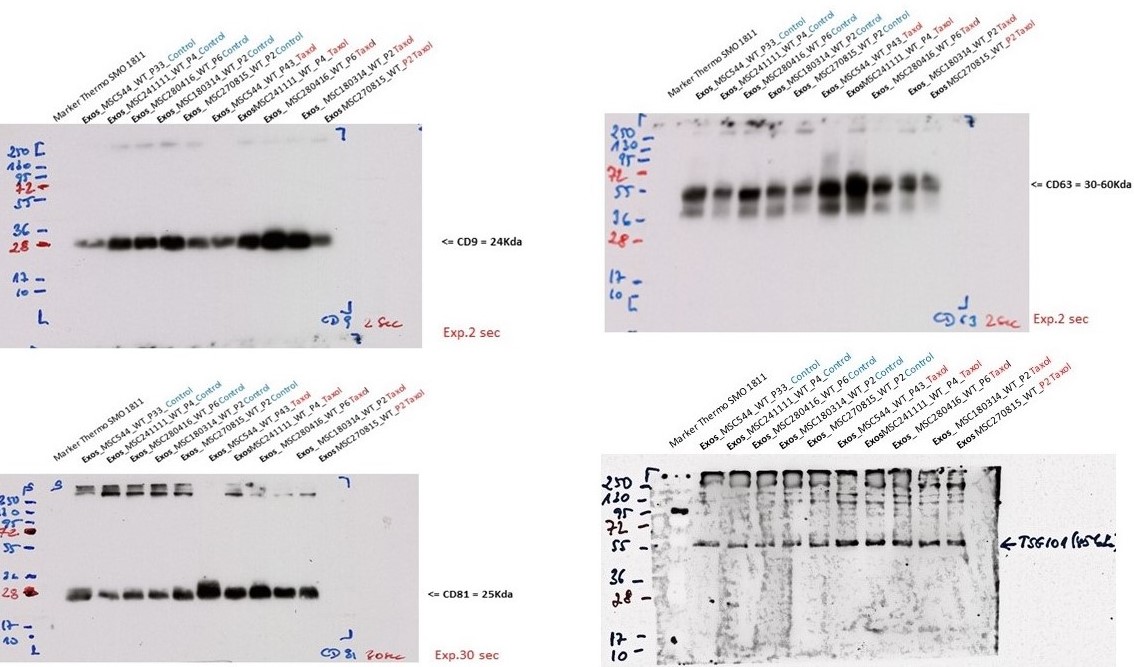

Supplement: Supplementary file 2 — Additional file 2. Supplementary Figure S2: Original Western blot data of MSC-derived EVs/exosomes. [file 12964_2024_1886_MOESM2_ESM.jpg]

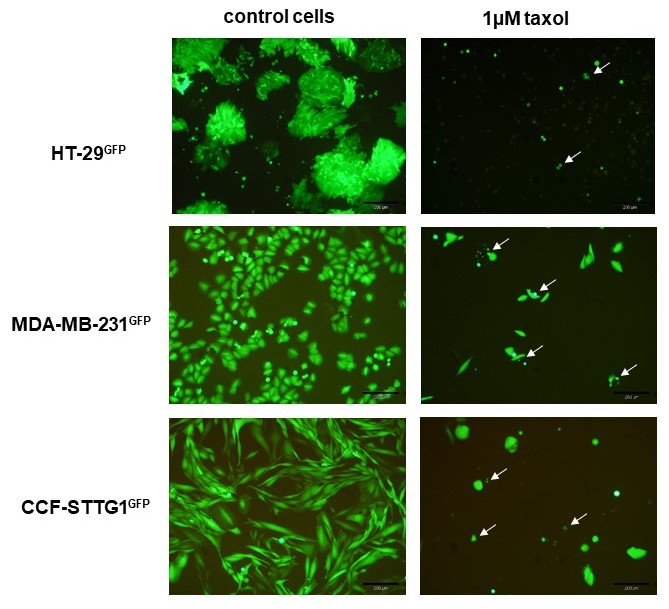

Supplement: Supplementary file 3 — Additional file 3. Supplementary Figure S3: Human MDA-MB-231GFP breast carcinoma were treated with 1µM taxol for 72h, and the slower proliferating HT-29GFP colon adenocarcinoma and CCF-STTG1GFP astrocytoma cells were exposed to 1µM taxol for 168h, respectively. Documentation of the cells was performed using a fluorescence microscope (Olympus IX50). Desintegration of cells and cellular debris are indicated by white arrows. Bars represent 200µm. [file 12964_2024_1886_MOESM3_ESM.jpg]

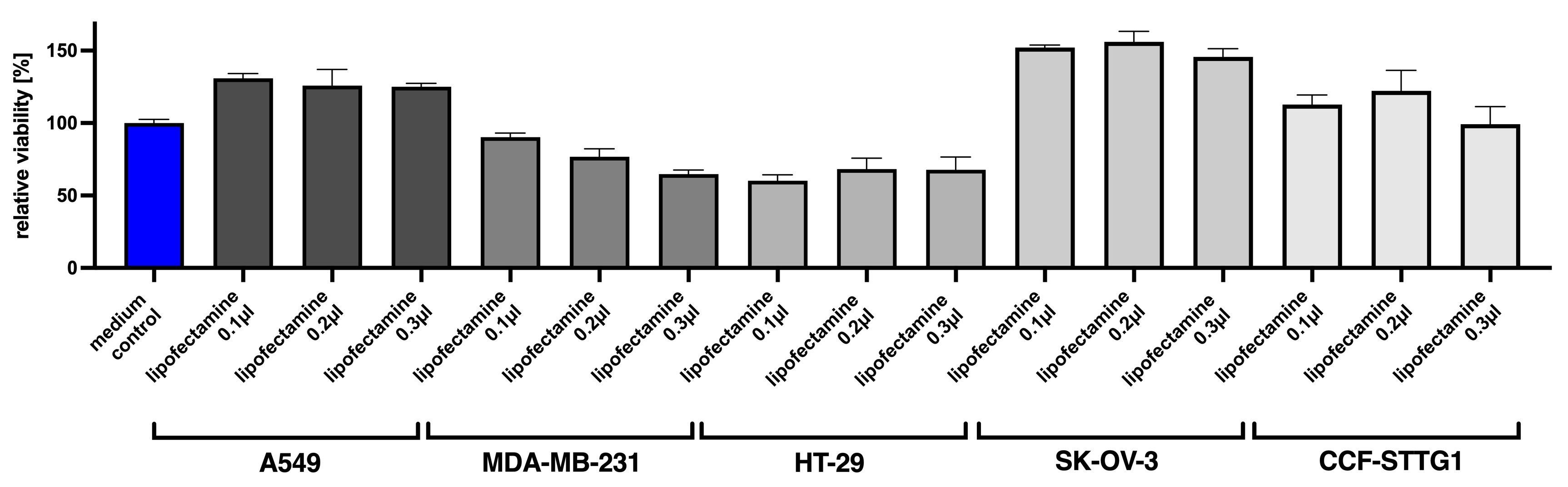

Supplement: Supplementary file 4 — Additional file 4 . Supplementary Figure S4: Lipofectamine concentration-dependency in different cancer cell lines. [file 12964_2024_1886_MOESM4_ESM.tiff]

Cluster analysis of differentially expressed miRs in EVs/exosomes

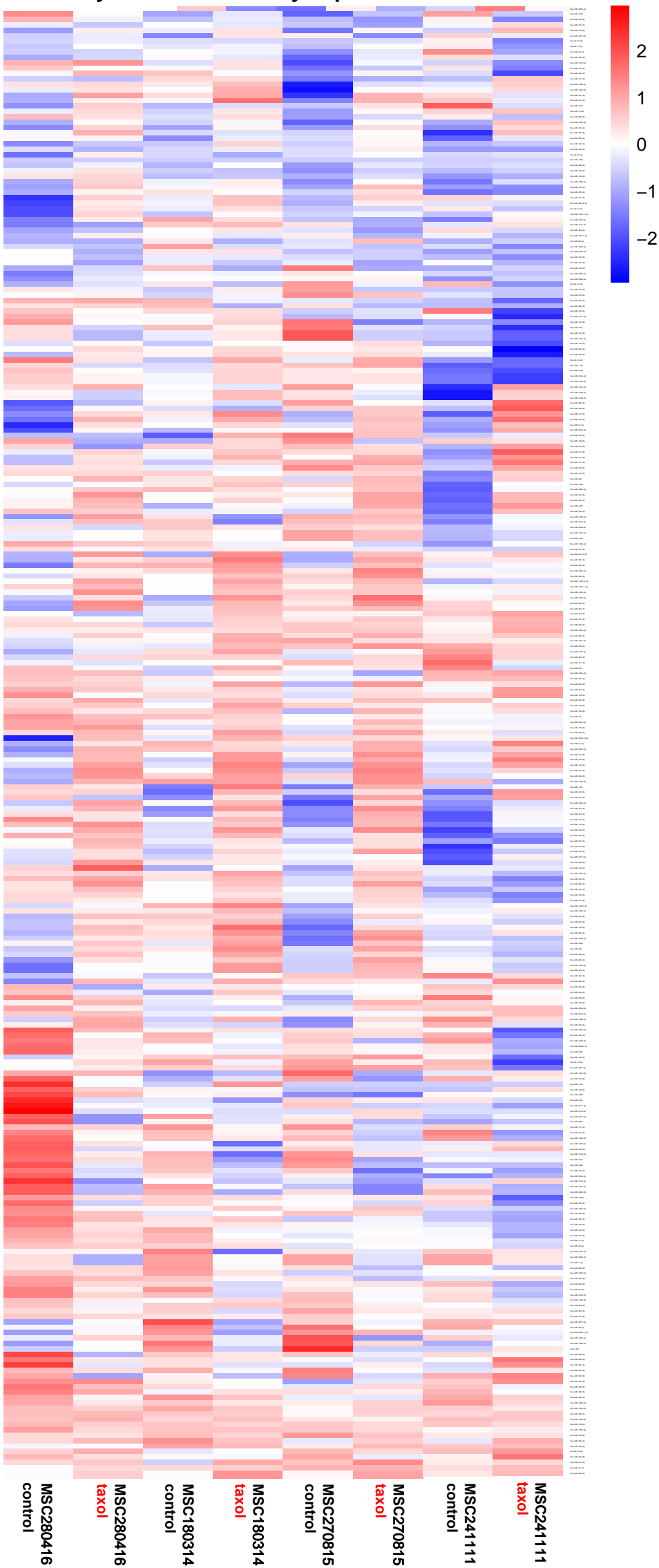

Supplement: Supplementary file 5 — Additional file 5. Supplementary Figure S5: Heatmap cluster analysis of miRs in MSC-derived EVs/exosomes and taxol-treated MSC-derived EVs/exosomes. [file 12964_2024_1886_MOESM5_ESM.pdf]

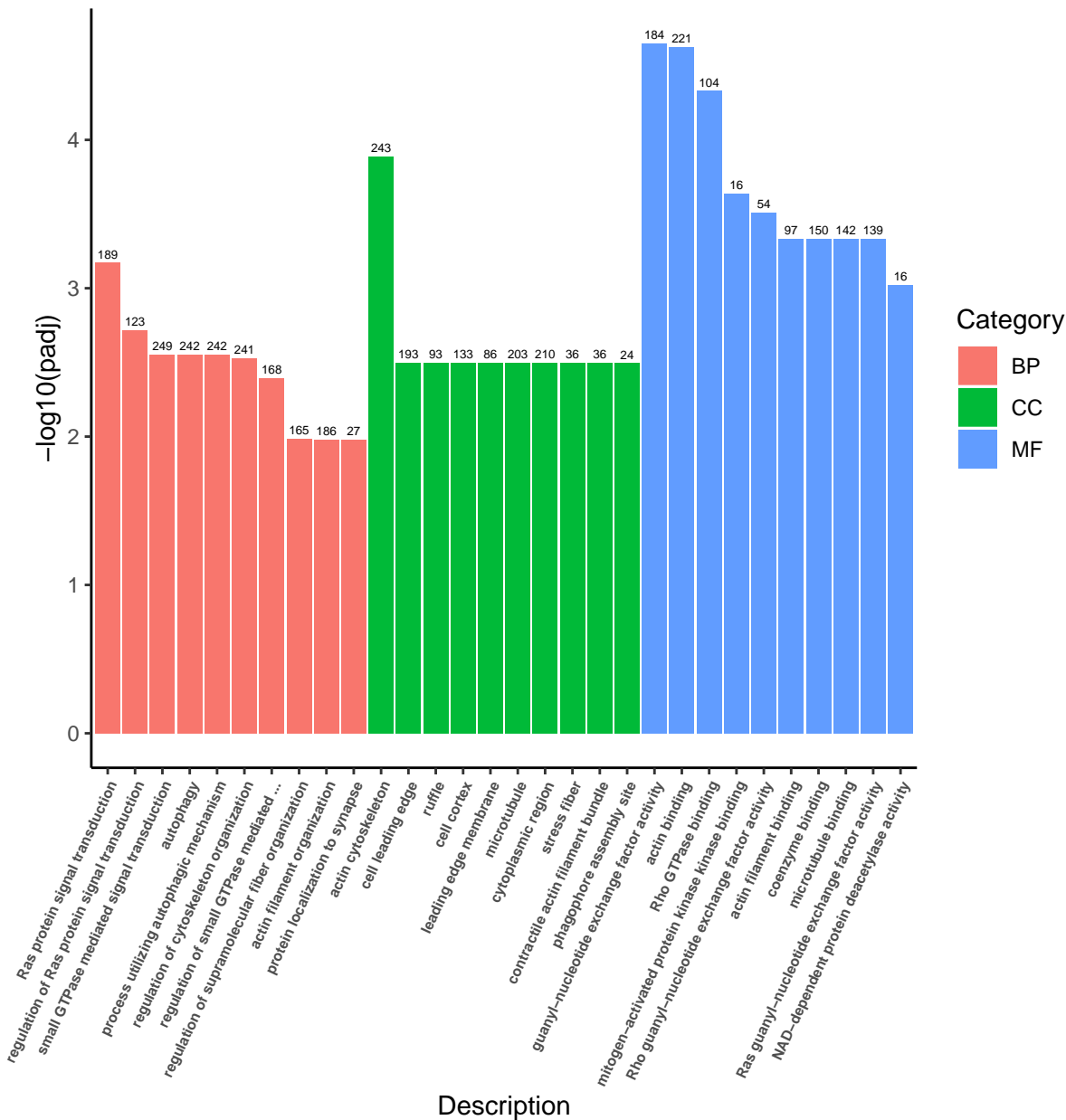

Supplement: Supplementary file 6 — Additional file 6. Supplementary Figure S6: Gene ontology (GO) analysis was performed in differentially expressed miRs of four MSC-derived taxol-loaded exosome populations (MSC241111 P4; MSC280416 P6; MSC180314 P2; MSC270815 P4) as compared to the corresponding control EV/exosome miRs of the four MSC. Pathways affected by the differential miR expressions were discriminated by biological process (BP), cellular component (CC), and molecular function (MF), respectively. [file 12964_2024_1886_MOESM6_ESM.pdf]

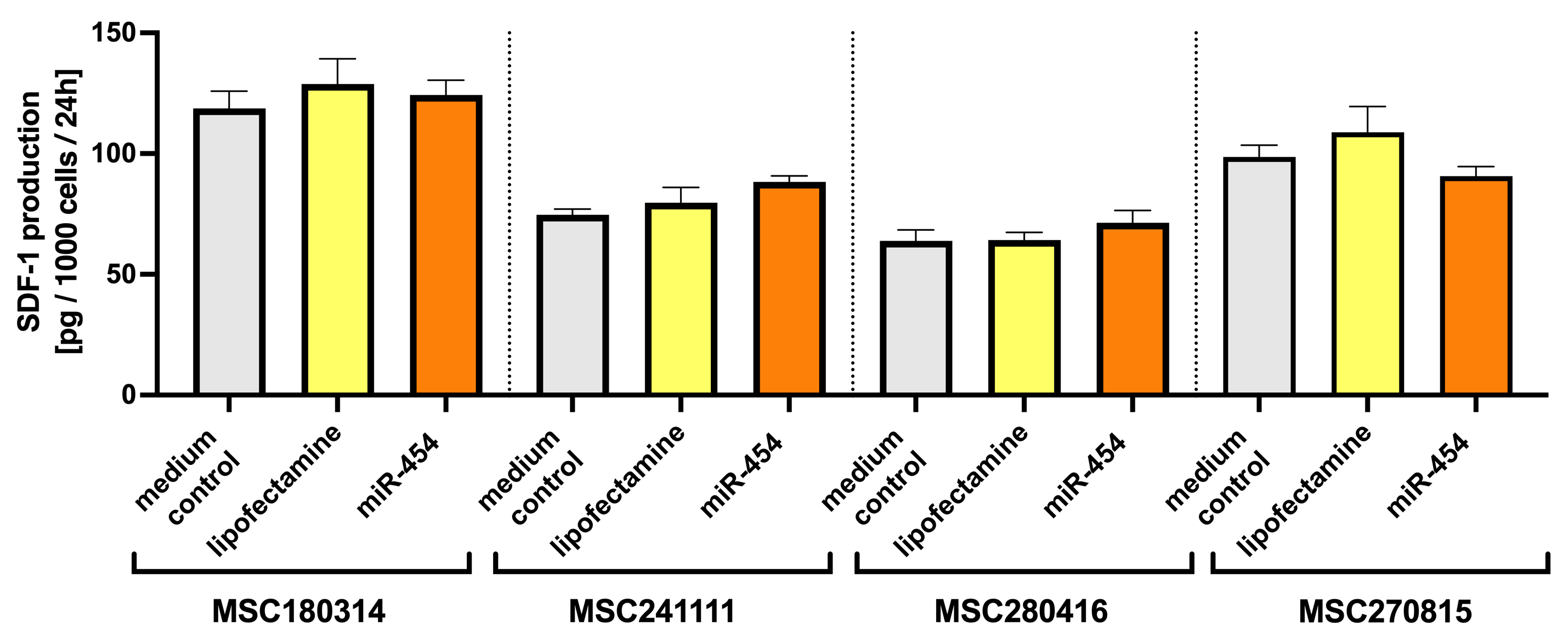

Supplement: Supplementary file 7 — Additional file 7 . Supplementary Figure S7: The four different MSC populations were incubated in culture medium (medium control), after transfection with 0.3µl lipofectamine (lipofectamine), after transfection with lipofectamine and a mixture of 2.5pmol miR-454-3p and 2.5pmol miR-454-5p (miR-454), and after stimulation with 10µM taxol (taxol). Following 24h of incubation the amount of released SDF-1 into the culture medium was quantified by an appropriate ELISA and calculated according to an equivalent amount of 1000 cells. Data represent the mean + s.d. of three independent experiments. [file 12964_2024_1886_MOESM7_ESM.tiff]

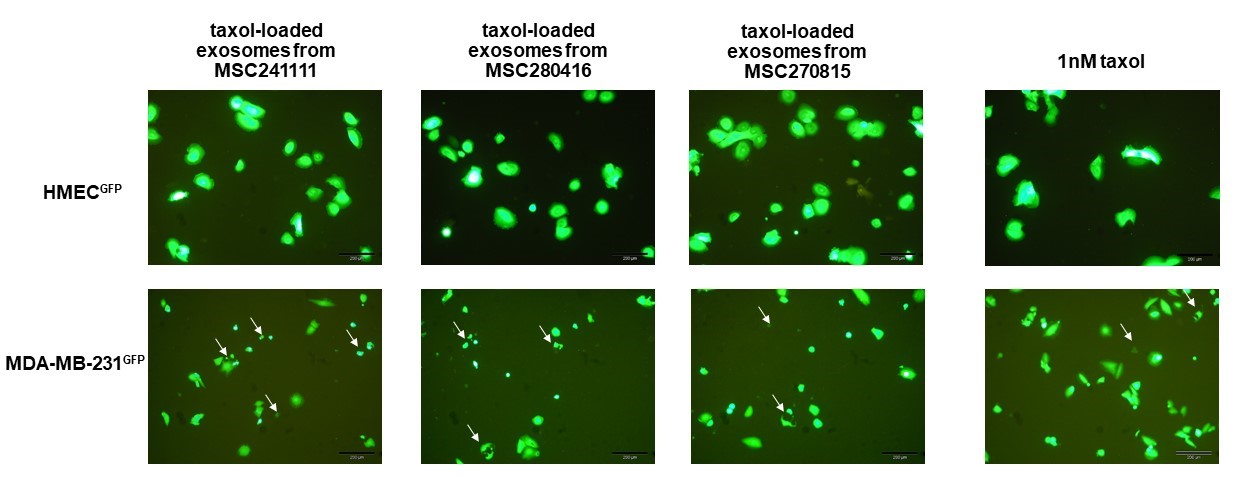

Supplement: Supplementary file 8 — Additional file 8. Supplementary Figure S8: GFP-labeled normal human mammary epithelial cells (HMECGFP) and human breast cancer cells (MDA-MB-231GFP) were treated with taxol-loaded exosomes isolated from three different human MSC populations (MSC241111, MSC280416, and MSC270815) in a 96-well microtiter plate for 72h, respectively. Incubation of the cells with 1nM taxol for 72h served as a control. Documentation of the cells was performed using a fluorescence microscope (Olympus IX50). Desintegration of cells and cellular debris are indicated by white arrows. Bars represent 200µm. [file 12964_2024_1886_MOESM8_ESM.jpg]
